# Myths and misconceptions of intimate partner violence among sexual and gender minorities: a qualitative exploration

**DOI:** 10.3389/fsoc.2024.1466984

**Published:** 2024-12-20

**Authors:** Stefan Kurbatfinski, Nicole Letourneau, Susanne Marshall, Dawn McBride, Jason Novick, Keira Griggs, Arielle Perrotta, Morgan Daye, Carrie McManus, Kendra Nixon

**Affiliations:** ^1^Department of Community Health Sciences, Cumming School of Medicine, University of Calgary, Calgary, AB, Canada; ^2^Owerko Centre, Alberta Children’s Hospital Research Institute, Calgary, AB, Canada; ^3^Department of Pediatrics, Cumming School of Medicine, University of Calgary, Calgary, AB, Canada; ^4^Department of Psychiatry, Cumming School of Medicine, University of Calgary, Calgary, AB, Canada; ^5^Faculty of Nursing, University of Calgary, Calgary, AB, Canada; ^6^Faculty of Education, Counsellor Education, University of Lethbridge, Lethbridge, AB, Canada; ^7^Departments of Anthropology and Archaeology, University of Calgary, Calgary, AB, Canada; ^8^Sagesse Domestic Violence Prevention Society, Calgary, AB, Canada; ^9^Rowan House, High River, AB, Canada; ^10^Faculty of Social Work, University of Manitoba, Winnipeg, MB, Canada

**Keywords:** myths, misconceptions, intimate partner violence, sexual and gender minority, qualitative research

## Abstract

**Background:**

Intimate partner violence (IPV), referring to different forms of violence or abuse between two or more intimate partners, negatively impacts physical and mental health, performance in various settings, and familial functioning, leading to long-term adverse outcomes. Sexual and gender minority (SGM) individuals tend to experience similar or greater frequencies of IPV compared to their cisheterosexual counterparts. Stigma and discrimination toward sexual and gender diversity can lead to myths and misconceptions about relationship dynamics among SGM individuals, which can contribute to IPV occurrence within the community. This study sought to: (1) develop a compendium of myths and misconceptions that SGM individuals exposed to IPV and relevant service providers shared they encountered; (2) describe the impacts of these myths and misconceptions on SGM individuals experiencing IPV; and (3) make recommendations to address these myths and misconceptions.

**Methods:**

This qualitative study used data from a larger project focused on SGM IPV where SGM individuals who experienced IPV (*n* = 18) and service providers who supported SGM individuals experiencing IPV (*n* = 8) were interviewed using semi-structured formats. Thematic content analysis and inductive approaches were used to identify and organize findings into themes and subcategories.

**Findings:**

Five major themes related to SGM IPV myths and misconceptions were identified, touching on aspects including, but not limited to, SGM IPV prevalence, prescribed gender roles and expectations, and societal factors. Impacts and recommendations are also discussed.

**Significance:**

This appears to be the very first in-depth study describing myths and misconceptions that SGM individuals and relevant service providers have encountered in regard to SGM IPV, helping to promote understanding of SGM intimate partner relationships with particular relevance to public health and social services policies.

## 1 Introduction

Intimate partner violence (IPV) includes physical, sexual, emotional, financial, legal, spiritual, religious, and cultural violence or abuse between two or more intimate partners (Doyle et al., 2022). More relevant to sexual and gender minority (SGM) individuals, including Two-Spirit, lesbian, gay, bisexual, trans, queer, questioning, intersex, and asexual individuals, among others, IPV also includes identity abuse such as gender invalidation (Woulfe and Goodman, 2021). IPV has been historically described in the cisheterosexual (cishet) context in which a cishet man engages in abuse or violence against a cishet woman (Hunnicutt, 2009; Patra et al., 2018). Such discourses may not characterize the experiences of SGM individuals and even create ambiguity and confusion as SGM relationships may no longer adhere to the traditional cishet male on cishet female abuse discourse (Workman and Dune, 2019). Since a substantial portion of individuals, both inside and outside of the SGM community, learn about IPV through the cishet discourse, a plethora of myths and misconceptions can arise when contextualized outside of the traditional representation of IPV (Rollè et al., 2018). These myths and misconceptions can be pivotal in IPV experiences, influencing the entire IPV journey from receipt or use of IPV to help-seeking behaviors (Rollè et al., 2018); ensuring that attention is brought to such narratives is imperative to promote clarity of SGM IPV and to disentangle false discourses which convolute IPV experiences.

### 1.1 The cishet relationship context

Stemming from a patriarchal, traditional discourse in which cishet men often exuded dominance and control over cishet women, IPV has historically been described as occurring solely between two individuals, where a cishet man engages in, and a cishet woman experiences, IPV (Hunnicutt, 2009; Patra et al., 2018). Such narratives sprout from socially constructed gender roles which dictate behavior and relational outcomes based on gender (McCarthy et al., 2018). For example, studies have elucidated how men may engage in more abusive behaviors when they endorse certain misconceptions such as women not needing to work if the husband is making a sufficient amount of income or that they have the right to sexual activities with their partner whenever they desire (McCarthy et al., 2018). Likewise, cishet women who endorse prescribed gender roles or misconceptions around IPV deservingness when defying expected gender roles are more likely to accept and normalize their experience (McCarthy et al., 2018; Overstreet and Quinn, 2013). While cishet women can also engage in abusive behaviors and exert dominance over cishet men within intimate partner relationships (Machado et al., 2024), it is well understood that the severity and frequency of IPV experienced by cishet women is much greater compared to cishet men (Holmes et al., 2019). For example, the rate of femicide, which is often perpetrated by an intimate partner, has increased by 27% from 2019 to 2022 in Canada with at least one girl or woman being murdered every 48 hours (Canadian Femicide Observatory for Justice and Accountability, 2024). A report conducted by Statistics Canada in 2023 using police-reported data further revealed that 29% of female homicides, compared to 4.2% of male homicides, were perpetrated by an intimate partner (assumed to include both cis and trans females and males) (Statistics Canada, 2024). For this reason, much of the IPV discourse continues to maintain a cishet male on cishet female context.

Myths and misconceptions can also cause impacts on help-seeking for individuals experiencing IPV. For example, women may be hesitant to seek support if they believe that the IPV they are experiencing is their fault or if they adopt cultural beliefs of IPV acceptance (Gurm and Marchbank, 2020). Supports may also blame, shame, and stigmatize women if they diverge from constructed gender beliefs and expectations (Overstreet and Quinn, 2013). Men who experience IPV may be reluctant to report their experiences as they develop self-internalized embarrassment, want to maintain a “masculine” persona, or attempt to align with typical cishet relationship standards (Taylor et al., 2022). Conversely, if men attempt to seek help, they may be denied support (Taylor et al., 2022). Also, some individuals may desire to identify new means of reducing abuse or violence in their relationship; however, if supports adopt beliefs that the termination of an abusive relationship is always the goal for individuals experiencing IPV, they may provide stigmatizing or suboptimal responses.

### 1.2 Dynamics within SGM populations

The SGM community is composed of multiple sexual and gender identities and groupings that diverge from the traditional cishet identity (Workman and Dune, 2019). Many SGM individuals may learn about intimate partner relationships in the context of a cishet relationship; however, as SGM relationship dynamics can differ greatly compared to cishet relationships, SGM individuals may overlook or minimize their partner’s behavior as abusive or violent (Rollè et al., 2018). For example, the downplay of woman-on-woman abuse causes challenges within lesbian relationships as partners minimize their experience of IPV with the belief that only cishet men can engage in such behaviors (Brown, 2008). The assumption that IPV can only occur between two partners who have a long-term relationship is also detrimental as it stigmatizes, distances, and downplays the experiences of sex workers, sexually active individuals, and polyamorous individuals (Zemlak et al., 2024). The conceptualization of bidirectional IPV also changes as SGM relationships may no longer consist of a cishet man and cishet woman; for example, there may be a magnification on physical appearance when providers are supporting SGM individuals experiencing IPV, especially in same-sex couples, which can lead to perceptions of “mutual conflict” (Kirschbaum et al., 2023; Rollè et al., 2018). In fact, studies conducted with SGM relationships show greater prevalence of bidirectional abuse compared to cishet relationships (Machado et al., 2024). All these examples show the harms that IPV myths and misconceptions can have on SGM individuals and the need to highlight and unlearn such understandings.

### 1.3 Purpose of the study

It is fitting to determine IPV myths and misconceptions from the experiences of SGM individuals affected by IPV and from relevant service providers (i.e., service providers working with SGM individuals impacted by IPV) to promote IPV disclosure and support seeking. Therefore, we aimed to develop a compendium of myths and misconceptions while answering the following research questions: (1) what myths and misconceptions do SGM individuals experiencing IPV and relevant service providers encounter regarding SGM IPV? (2) what are the impacts of these myths and misconceptions; and (3) what recommendations can help to reduce myths and misconceptions to better support SGM individuals experiencing IPV? To answer these questions, we used semi-structured interviews with SGM individuals who experienced IPV and relevant service providers.

## 2 Methods

Data for this secondary qualitative study analysis are derived from a mixed-methods study conducted by the Research and Education for Solutions to Violence and Abuse (RESOLVE) Network which aimed to examine the context of IPV among SGM individuals who experienced IPV and relevant service providers (Haller et al., 2022). More specifically, this larger report sought to elucidate how IPV was experienced by SGM individuals across Alberta, Saskatchewan, and Manitoba, the three prairie provinces in Canada. Alberta, the setting of this study, is located in western Canada with high rates of gender-related homicide among women and girls (Sutton, 2023). We followed the Consolidated Criteria for Reporting Qualitative Research (COREQ) guidelines when reporting this research (Supplementary material Table A) (Tong et al., 2007). We obtained ethical approval from the University of Calgary Conjoint Health Research Ethics Board (REB20-1461_REN3).

### 2.1 Participant eligibility and data collection

Eligible participants included self-identifying SGM individuals who were at least 18 years of age, experienced IPV within the last 10 years, resided in a western Canadian province during their experience(s) with IPV, and were no longer living with their abusive partner. Participants were recruited through posters placed in libraries, community centers, and service provider agencies (upon voluntary agreement) and through social media and university website posts. Service providers eligible for inclusion worked for an organization providing services to SGM individuals experiencing IPV. Following an environmental scan of existing services, the research team directly contacted these organizations to engage service providers as participant interviewees.

Semi-structured interviews were conducted by telephone, and questions were provided in advance to reduce interview anxiety if desired. Prior to starting the formal interview questions, interviewers provided a brief rationale for the purpose of the study. Participants were asked questions about their demographics, types of IPV experienced, perceptions regarding the intersection between their sexual and/or gender identity and the IPV occurrence(s), help-seeking experience with formal and informal supports, and broader perceptions of SGM IPV within society (Supplementary material Table B) (Haller et al., 2022). Interviewers (JN, SM, SK, OG) recorded interviews via a hand-held recording device and verbally collected participants’ demographic information at the start of each interview. All interviewers had at minimum an undergraduate degree in social sciences or biological sciences. Debriefs were offered to all participants following interview completion and we provided a list of no-cost supports if any participants required them. Interviews ranged from 25 to 135 min in length. After the interviews were completed, interviewers immediately uploaded audio files to a secure online drive with a two-factor password encryption system, removing and destroying the audio files on the recording devices. Interviews were transcribed manually and with Otter.ai, an online platform which uses artificial intelligence to transcribe audio files (Otter.ai, 2019). We also manually reviewed the accuracy of the transcripts completed through Otter.ai (2019). While we encouraged participants to refrain from using identifying names during interviews, we also removed any potentially identifying information from transcripts. To maintain anonymity, transcripts were labeled only with participant identification numbers. We provided an honorarium ($40 CDN) to participants who experienced IPV.

### 2.2 Data analysis

Three authors (SK, JN, SM) performed a thematic content analysis of the interviews to identify and report themes related to myths and misconceptions (Braun and Clarke, 2006, p. 79). Dedoose, a web application designed to facilitate qualitative research, was used to manage and analyze all qualitative data (Dedoose, 2018). A focus on myths and misconceptions as an overarching concept occurred after completing all the data analysis phases from the larger report. Given how difficult it is to locate peer-reviewed literature that provides a comprehensive list of SGM IPV myths and misconceptions, we wanted to investigate this in more detail from this dataset and create a compendium of myths and misconceptions. We used inductive approaches to identify major themes. The same three authors then identified subcategories that best organized the myths and misconceptions under the developed major themes. More specifically, two authors (JN, SM), who also conducted the interviews, first identified patterns in the data and a third author (SK) reviewed all the interviews and analyses to enhance rigor by reducing subjectivity; these three authors met regularly to discuss findings and reach a more objective consensus. Data saturation was achieved by iteratively examining the interviews and organizing findings under themes and subcategories.

The SGM group included diverse genders, sexual orientations, and socioeconomic groups, which we used to contextualize and differentiate myths and misconceptions encountered by different groups while preserving participants’ privacy and confidentiality. For theme 1, myths and misconceptions applying more generally to the SGM community were grouped under different categories based on similarity in content. For theme 2, myths and misconceptions were categorized based on each respective SGM identity group, when possible. For theme 3, myths and misconceptions related to other types of identities beyond sexual orientation and gender were grouped based on similarity in content. When possible, we also discussed how overlapping identities (e.g., sex and race) affected individuals’ experiences with IPV myths and misconceptions.

### 2.3 Meaningful engagement

To facilitate meaningful engagement, we invited service providers to review the entire draft and contribute to the discussion section of this paper as authors; five service providers helped to prepare this manuscript by writing portions of the discussion and reviewing the entire manuscript. We met online to discuss the manuscript and to identify any barriers that existed in terms of the transparency and dissemination of IPV research.

## 3 Findings

Participants in this study (mean age 28.94 years ± 6.99) mostly self-identified as white/Caucasian (*n* = 11, 61%), their sexual orientation as lesbian (*n* = 9, 50%), and their gender as “female,” self-reported (*n* = 6, 33%; Table 1). Most SGM individuals experienced emotional or psychological violence or abuse (*n* = 16), followed by physical (*n* = 11), sexual (*n* = 10), stalking (*n* = 6), and financial violence or abuse (*n* = 5). All eight service providers self-identified as a woman and worked in director or coordinator roles for 5 years or less; half (*n* = 4) worked in agencies providing SGM-specific support through direct services, while the remaining four worked in shelter (*n* = 2), police-based (*n* = 1), or sexual assault center (*n* = 1) services. SGM individuals commonly discussed their experiences of IPV in relation to their sexual and gender identity, contextualizing their experiences on feelings of shame, stigma, and oppression and the consequent barriers that arose when trying to seek help.

**Table 1:** Demographic information about SGM individuals experiencing IPV (*N* = 18).

Demographic variable	Number of participants (#)
Race	
White	11
Asian	2
Indigenous	3
Biracial	1
Hispanic	0
Not answered	1
Sexual orientation	
Lesbian	5
Gay	1
Bisexual	5
Pansexual	1
Other*	6
Gender	
Female	4
Male	1
Transman/male	2
Transwoman/female	2
Cis/cisgender/cisgender female	2
Two-spirit	1
Genderfluid/queer	2
Other*	4
Education	
High school	0
Some college or undergraduate school	7
Undergraduate degree or college diploma	10
Graduate degree	1
Annual household income CAD dollars	
$0–$24,999	6
$25,000–$59,999	5
$60,000–$99,999	4
$100,000–$200,000	2
Not answered/did not know	1
Employment status	
Unemployed	5
Student	1
Employed part-time or contract	3
Full-time	9

Other* includes individuals who described two or more sexual orientations or gender, respectively.

Throughout their narratives, SGM individuals experiencing IPV and relevant service providers alluded to many myths and misconceptions that they encountered when experiencing IPV or in their practice (in addition to direct questions related to myths and misconceptions), respectively, highlighting the importance of this study. Overall, we categorized findings under five major themes, including (1) myths and misconceptions about IPV more generally and its prevalence, (2) IPV myths and misconceptions in relation to gender and sexual identity, (3) societal factors influencing myths and misconceptions and IPV experiences, (4) impacts of myths and misconceptions of gender and sexual identity, and (5) recommendations and areas for improvement. A more exhaustive list of myths and misconception quotes that support the major themes and their subcategories is provided (Supplementary Material Interview Guide).

### 3.1 Theme 1: myths and misconceptions about IPV more generally and its prevalence

*IPV Understandings in Society more Generally (n=7):* Seven participants described myths and misconceptions about cishet man-on-woman IPV that they encountered in society more generally as captured by the following quote: “[people think] a girl cannot do as much damage to another girl or a man cannot do as much damage to another man. Like, that’s the biggest misconception out there” (P1). Participants also described “how relationships, in general, are portrayed” (P2) and how misperceived “standards of how people in a certain gender should act” create misconceptions within SGM relationships. (P3). One participant (P17) alluded to the overarching idea captured by these quotes, such that “attacking the gender identity” shifts the focus from providing support to creating assumptions and minimizing incidents of SGM IPV.

*Commonality of IPV (n = 8):* Participants spoke on how there seems to be an underestimation of IPV prevalence within the SGM community. For example, one participant expressed that they “do not think people even know how common [IPV] is” (P5), reducing the gravity of the situation. The underestimation may also stem from not perceiving incidents as IPV: “a lot of people in the community have been victims of emotional abuse without even actually realizing that that’s the case” (P7). Individuals within the SGM community may “just laugh at it, or they are like ‘this is fine’, but it’s really not fine… Basically people need to acknowledge that it’s not ok” (P14). A participant also expressed how “there’s a certain expectation within the community that like we have the world by the tail… we cannot let anyone know that we are struggling. We cannot go to the straight world and tell them that we are, you know, experiencing the same things that they do” (P8). Another participant paralleled this idea but stated that they “would not say that people who are part of the community are more prone to being abusers,” but that the attempt at maintaining a “welcoming, friendly, happy, loving community [image]” makes it “get underreported or minimized” (P17). Overall, preserving the image of the SGM community is “kind of, like, a cloak and dagger when it comes to trying to divulge issues. But were humans, everybody has issues” (P8).

*Healing is not Uniform (n=2):* There is at times a misconception that an individual who has experienced IPV cannot heal postseparation. Two participants provide statements of hope in respect to healing following the end of an abusive relationship: “just because you have been through a situation of intimate partner violence does not mean that you are unable to be stable and healthy in a relationship… and that you cannot be resilient and strong” (P7) and “you are able to do okay after, to work hard” (P16). These participants express that people can overcome their experiences of IPV and enter healthier relationships in the future.

### 3.2 Theme 2: IPV myths and misconceptions in relation to gender and sexual identity

*Gay Individuals (n = 6):* The manifestation of myths and misconceptions among gay men was discussed. This was exemplified by denial or minimization of the prevalence of IPV among gay men with respect to their physique: “stereotypes around ‘oh, but you are the stronger one’ can apply in homosexual relationships where it’s like ok but you two are, like, quote unquote, equals” (P13). This also alludes to notions of masculinity versus femininity. One participant stated how “if there’s like, one more, more butch, and one more feminine, [the IPV would] be taken seriously [by professionals]” (P16) compared to a gay relationship where partners share more similar qualities. Another participant extended this through discourses regarding tops (those who prefer/only enjoy anally penetrating another individual) and bottoms (those who prefer/only enjoy being anally penetrated): “also for like tops who have been sexually assaulted, I think a lot of people are like how is that even possible? I thought you would have to be… bottoming for that to happen” (P4). Tops are often assumed to be dominant, while bottoms are associated with submission, triggering a constructed masculinity complex that may feel like it needs to be upheld even if violence or abuse is experienced. This same participant also expanded on myths regarding sexual violence, such that there is a conceived notion that “gay men always want to have sex” and “experiencing an erection while being sexually assaulted” must be reflective of enjoying the experience. This neglects the difference between physical versus mental stimulation and lessens the gravity or severity of the assault. In relation to this, another participant described how there is “some level of [non-consensual] normalized sexual touching in the gay community” (P13). Lastly, one participant alluded to the judgmental and preconceived ideas that many people might have regarding a gay relationship if it aligns with socially constructed perceptions such as an “open, non-monogamous [relationship with lots of] part[ies], and you know, [having] drugs involved, and HIV” or “[those using] a bathhouse” (P15).

*Lesbian Individuals (n = 8):* A prominent misconception among lesbian individuals was that women cannot be abusive in lesbian relationships, whereby a non-binary, Two-Spirit individual described misconceptions that they have encountered in society: “how do you get raped by a woman” or “how can someone be raped if there’s no penis involved kind of thing” (P18). Another individual stated that people may be “so dismissive as to think that between two women you cannot have sex” (P8), eliminating the potential for sexual violence to occur among women-only relationships altogether. This participant also shared how their doctor “looked at [her following disclosure of abusing and said] ‘you are fine’.” The extent of impact is further contextualized in the context of physical and emotional lesbian IPV: “when a girl hits a girl, it’s not [viewed] the same… a woman cannot do the same kind of damage as a man can. But they can, emotionally and physically” (P1). This extends into myths about understanding lesbian IPV, whereby “lesbian intimate partner violence… [is] not seen as, as legitimate” (P19) and that others “just do not really know what to expect” (P16). Similar to gay individuals, there was also a misconception that lesbian partners had to embody certain roles in the relationship: “[you are] either like the home type of lesbian or you are the party lesbian” (P7) and “playing like [being] more butch and [being] more tough and [being] more hard and just like more masculine, I think that also affects how they portray themselves” (P16). Two participants also described the UHAUL myth, which suggests that “lesbians [tend to] immediately move in together” (P7). Additionally, normalizing the “expectation of emotional disruption in lesbian relationships” (P7) ultimately downplays lesbian IPV.

*Trans Individuals (n = 6):* Gender stereotyping concerning the perpetration of IPV within trans relationships was also exemplified. Transphobic narratives persisting in society have perpetuated a stereotype that trans individuals are violent or dangerous; one participant stated how the occurrence of IPV among trans individuals “just kind of keeps with that stereotype that a lot of trans folks are trying to fight against that you know, they are like violent or dangerous” (P9). Further, gender constructs manifested uniquely for trans individuals, whereby one participant described how they “identified as a trans female now,” yet other individuals, such as “the police,” may still consider them to be a man, suggesting that they “should be able to handle it and take it [the IPV]” (P6). Another trans man affirmed this idea, such that they were “met with a lot of incredulous looks [when seeking support], especially as a trans man because then it’s not girl on girl abuse” (P3). On the other end, some participants encountered the misconception that “transmen cannot be misogynistic” (P17). This reveals misconceptions pertaining to not only trans identities but also represents how misconceptions around men needing to endure violence and that women cannot be abusive trickle in and convolute SGM IPV experiences. These intentional or unintentional misconceptions also result in identity abuse toward trans individuals, whereby one trans non-conforming participant stated how “like, I’ve even had people tell me like, well, you have the right parts [of a woman’s body]” (P18), fully delegitimizing their gender non-conformity. One participant sheds light on the fact that though transphobia is prevalent outside the SGM community, it can also occur within the community: “trans-femmes can be discriminated against in woman and non-binary spaces because they are still flagged, even sometimes in our own community, as men which is disgusting but happens” (P19).

*Two-Spirit, Bisexual, Queer, Questioning, Polyamorous, Pansexual, and/or Non-Binary Individuals (n=7):* Authors grouped Two-Spirit, bisexual, queer, questioning, polyamorous, pansexual, and non-binary individuals due to the smaller number of quotes in relation to these identity groups. However, almost all spoke on how their sexual and gender identities are misunderstood by partners and others, complicating their experience with IPV. The portrayal that “queer couples are automatically healthier than straight ones” (P18) was a myth repeated by three participants, describing how society perceives queer relationships. Conversely, another described a myth that they have encountered pertaining to queer IPV prevalence such that “if you are sexually abused, you are more likely to be queer, and, or a part of the 2SLGBTQ+ community” (P13). A bisexual individual described how they “tried to disclose [their bisexual] identity to [a counselor] and she just told me I was just wrong and confused cause I’d just gotten out of this relationship” (P9), invalidating bisexuality as a sexual orientation altogether. A questioning/pansexual/bisexual individual described how their women-identifying ex-partner mentioned that “if you actually hated men you would identify as a woman… you should identify as lesbian only” (P17), dictating how their partner should be based on their gender and/or sexual orientation. This continued post-separation, such that the ex-partner mistook a photo of her brother for a new boyfriend which led to gender and sexual orientation invalidation: “you were never actually like bisexual, pansexual, you are just like faking it for attention” (P17). Both quotes demonstrate a misunderstanding of the ex-partner regarding what pansexual, bisexual, or questioning identities entail and disregard the potentially fluid nature of the participant’s sexuality and gender. Another described how service providers may not view “a non-binary abuser… as legitimate” (P19) while a pansexual participant described how police misunderstand and stigmatize polyamory: “well, what do you mean you have got 4 partners or 12 partners?” (P15).

A service provider also alluded to the misconception that society maintains around gender and sexuality being static, such that “we tend to assume that everybody is knowing [their gender and/or sexual orientation] from the time that they are children, and therefore, we also tend to assume that gender and sexuality are static” (SP3). This individual further extends this notion by describing how “just because on the outside the person does not look like they are part of the community does not mean that they are not part of the community because there’s a lot of… identities under the umbrella of 2SLGBTQ+ that are often really overlooked” (SP3).

### 3.3 Theme 3: societal factors influencing myths and misconceptions and IPV experiences

*Race (n = 4):* Participants also spoke to the misconceptions in relation to race and IPV without necessarily contextualizing the misconceptions as manifestations of their sexual or gender identity in conjunction with their racial identity. For example, one white participant “felt like if [the police] did take [their disclosure of IPV] serious, they’d take it too serious” as their ex-partner was Indigenous and police often maintain discriminatory and colonialist attitudes toward Indigenous individuals (P10). This same person described how stereotypes about violence within certain cultures may convolute IPV experiences: “my ex was like upper tier, wealthier Chinese and I know some stereotypes would say well that’s what the culture’s like…,” excusing abusive behaviors based on cultural upbringings (P19). A non-binary, Two-Spirit individual shed light on this when their partner would threaten to call the policy if they defended themselves during abusive incidents: “he’s a white man. He knew that the police were gonna listen to him and not me” (P18). One cis gay white participant described how the “hyper-sexualization of Black men” and the “feminization of Asian men” within the gay community can be damaging for individuals within gay relationships if they do not align with these narratives (P4). Gay men of color must not only navigate their sexual preference already as a gay man, but must also navigate harmful myths and misconceptions about their prescribed sexual roles which can put pressure on them, cause cognitive dissonance, and lead to undesired sexual activities.

Lastly, one non-binary, Two-Spirit Indigenous individual described how in addition to individuals outside the SGM community, individuals in the SGM community (with an emphasis on white individuals) may contribute to misconceptions and racist attitudes in relation to Two-Spirit and Indigenous identities: “There’s so much misinformation, right?… Like, even people within the queer community… People are just like, LGBT.’ And I’m like, ‘you actually left out Two-Spirit.’ And people are like, ‘well, that does not matter’…‘oh, well, that’s just too confusing…’ And they are like, ‘well, I’m trans, and I have to deal with this stuff.’ And I’m like, ‘yeah, but you are a white trans person, you still have so much work. Yes, you are part of a community, but that does not exempt you from also, like hurting the community’, you know?” (P18). It touches on the fact that though SGM individuals experience discrimination and marginalization, they are not exempt from supporting other SGM individuals who experience compounding oppressions due to society’s response to their sexual orientation and/or gender and their race.

*Religion (n = 2):* Misconceptions pertaining to IPV among sexual and gender minority intersected with religion for two participants. One described how “growing up in an environment with such a strict understanding of the binary of, like, gender and sex” convoluted their experiences of IPV (P13). It upheld patriarchal standards such that “women are blamed for being victims of sexual violence” (P13), demonstrating the implications of growing up within the powerful ideologies of religion. There were also misconceptions in religious environments that “if you are sexually assaulted or sexually abused, that will shape your sexuality or gender in some way toward being a part of the LGBTQ+ community,” suggesting that SGM individuals can be abused into their sexual or gender identity. Another participant described how their transphobic parents’ strong religious views shifted the narrative from focusing on the abusive nature of the relationship to “not [being from] the same religion” as a reason to end the relationship (P19), ignoring the IPV this participant experienced altogether and reflecting their parents’ disapproval of the abusive partner’s religion and/or gender identity rather than the IPV.

*Body Image (n = 7):* Seven participants spoke about the association between body image and IPV occurrence in the context of SGM relationships. This misconception pertained to erroneous perceptions among formal supports and society regarding larger body size as a protective factor against IPV. For example, “bigger guys, if they experience sexual assault, [other] people might say ‘how did someone like overpower you?’,” revealing a societal “narrow idea of what sexual assault is [where] someone bigger [is] like pinning a smaller person down” in the context of gay relationships (P4). Another participant mimicked this, such that their ex-partner was “very skinny and everything else” and so others would ask “how is she abusing you?”; this participant also emphasized that “it’s not always about physical, it could be mental” (P6). Others described how IPV can be regarded as unusual when partners have similar body images: “like the misconception thing would be just like appearance, judging by appearance. And like, yeah, I’m realizing that both partners are capable, even if they are very similar” (P16). This participant also described how they were judged more broadly by their appearance: “you do not seem like the regular person” (P16). One participant focused on the connection between age and SGM identity: “even if you are a bigger older person, you can still be hurt and violated” (P15). Lastly, one participant alluded to how there are certain societal constructs of what a SGM individual should look like, and because they “do not visually present as a member of the community,” it’s “not something that people automatically assume,” which can actually serve as a protective factor when disclosing IPV (P7).

*Conceptualizing IPV (n = 7):* Several participants encountered myths and misconceptions about what constitutes IPV. Most notably, participants discussed how emotional IPV is usually deemed less credible than physical IPV, such that if “it is not physical people do not really believe that” (P2). Others alluded to how they had their experience of IPV downplayed: “‘oh you were not, like, physically abused, like, she never hit you’, but emotional abuse is, like, almost deeper than that” (P14). A service provider reiterated this by describing how their clients would minimize their experiences of emotional abuse: “‘I wish they would just hit me’ or things like that because then ‘the people that were closest to me would actually understand that these things are happening to me and the impact that they are having on me’” (SP5). Participants also encountered misconceptions that society held toward what constituted an intimate partner: “people think of like, abuse in a relationship, as very like back in the 50s, of [man] beats wife. Man demands all of these things” (P18).

*Other Social Constructs (n = 10):* Participants also discussed myths and misconceptions that reflected social misunderstanding in relation to IPV more broadly. For example, several participants encountered misconceptions that “if the person looks like they are kind or they are generous” (P18) people will make statements such as “there’s no way, like, she’s too nice” (P1) or “that could not be true they knew this person and thought he was, well like a great person” (P9); these myths and misconceptions protect those engaging in abusive behaviors. Other participants described how people would question why they did not leave the abusive relationship earlier, suggesting that abusive partners are chosen or evident and completely ignoring the manipulative and emotional techniques often employed by those engaging in abusive behaviors. Two participants captured this misconception well: “sometimes people are really, really good at manipulating others. And it is an awful skill. But it’s a skill that they have. And it means that most of the time until it’s too late, you are not going to realize that you are being abused” (P2) and “sometimes it’s awful hard to see” (P19). Two participants described how people might say “that’s just who she is, or you just have to adapt your expectations a little bit” (P7), implying that abusive individuals cannot change.

Service providers drew some parallels in terms of myths and misconceptions described by SGM individuals who experienced IPV. For example, one service provider stated how SGM partners in an intimate relationship may themselves have misconceptions surrounding the nature of their own relationship: “the definition that the two people had was not the same even, so we have to get to the point where we are asking and allowing people to give an honest answer” (SP1). Another service provider also discussed the intersections between IPV and substance use and mental health challenges, such that their abusive partner could be “making them use substance in order to you know, support their own addictions,” rather than it being an independent choice (SP7). This is important to consider as service providers may hold misconceptions that SGM individuals want to engage in substance use rather than it being through involuntary consent, affecting how care is delivered.

*Consent (n = 3):* Myths and misconceptions pertaining to consent were also discussed by participants, manifesting differentially. One participant described how individuals might struggle to grapple with their experience of sexual abuse and violence if they were “consenting to aspects of it, but not all of it,” such that “when there’s a level of consent but not consent to everything” the occurrence of “sexual experiences [may occur] in the grey areas of consent” (P4). This was emphasized in the context of “hook-up culture,” where they described the misconception that “a lot of people who have kinky sex are like pros at consent” (P4), potentially misleading individuals to think that individuals with kinks are always good at providing or acknowledging consent. This participant also elaborated on how “non-physical ways of like coercing someone to do something that they are not completely ok with” are “not really recognized” (P4), such that consent is often considered only in the context of physical or sexual behaviors.

Another participant expressed the implications of Evangelical religion on understanding consent, such that their religion suggests that “women are sexual objects and do not, like, require consent” (P13). This participant also described how “by not leaving [the abusive relationship, they were] almost consenting to” the IPV they experienced, revealing an interesting perception of consent that is rooted in religious ideology. The last participant also described the idea of “giving in [to sexual stuff] cause [their abusive partner] will not stop” (P15), reinforcing the idea of pressured consent rather than voluntary consent. This participant also alluded to misunderstandings about consent at bathhouses and the minimization of the gravity of lack of consent when occurring in such environments.

### 3.4 Theme 4: impacts of myths and misconceptions of gender and sexual identity

*From the Experiences of SGM individuals who Experienced IPV:* Several important impacts resulted from the perpetuation of IPV myths and misconceptions within the frameworks of gender identity, sexual identity, and other societal factors. Almost all participants reported that they were reluctant to report IPV to formal and informal support due to a history of receiving facetious, invalidating, or disrespectful responses. Nearly half of these participants emphasized their apprehensiveness about submitting reports to the police specifically since they would not “really even get me, like, me or my body or my experience either” (P18). One participant also stated that they “had to go to court and obtain an emergency protection order to have the police” intervene in their IPV experience (P1). This extended into the medical sector, such that a participant knew a gay male individual who “went to the hospital with like, swelling on his face and huge bruises on his body, and said, ‘my boyfriend hit me, and he tried to choke me’. And the nurses did not take it seriously” (P16), resulting in suboptimal healthcare provision. One participant also demonstrated the implications of misconceptions about what constitutes IPV in the context of lesbian relationships, avoiding police “because it had not escalated enough that I was coming in with broken bones or bruises but I needed to talk to somebody” (P8), revealing (1) an internalized minimization of the emotional IPV they were experiencing and (2) a perception that police would disregard non-physical abusive and violent behaviors as forms of IPV. This same participant decided to go to their family doctor instead; upon disclosure of their abuse, the doctor responded as follows: “she looked at me, she’s like ‘you are fine’.” Overall, it seemed that “being in a LGBTQ relationship made it a little more difficult for [supports] to kind of take it seriously, right?” (P17). A participant further emphasized the impacts of considering only physical IPV as serious, such that “if people do not see physical evidence, they do not believe you” (P2). Another explained how misconceptions pertaining to what IPV is may cause people to “stay in those relationships longer or [to] assume it’s love” (P19).

One participant also discussed the implications of media on IPV: “kids do not learn about healthy relationships… based on what people are seeing in media, they, they people will try and base it off of that and, you know, it’s horrifically inaccurate, and it’s something that they literally do not know” (P2); ultimately, “when you are not taught anything else, you expect that to be how you are treated.” More specifically, another participant described how “we have all seen abuse in straight couples on screen,” convoluting how IPV is conceptualized among SGM individuals and reducing the likelihood of recognizing “abuse until it’s happened to you, and someone has straight up said to you, ‘hey, that was someone abusing you’” (P3). In relation to gay couples, the top versus bottom dynamic impedes on reporting experiences of sexual violence. The idea that a top could be sexually assaulted was described as “emasculating for some people depending on their gender identity,” preventing them to “come forward with experiences of sexual violence” (P4). This person also elaborated on how (1) “society might perceive [bathhouses]” and (2) society might understand “violence and consent as it relates to kink,” likely incurring stigmatizing responses which reduce desires to seek help. One person spoke on how “[the UHAUL lesbian myth] can be detrimental because people who have a good fit together might delay merging their lives based on that, or, it might have the opposite effect, it might be that like people assume that they have to move in together right away because of that” (P7). In relation to religion, a participant expressed how not fitting “into the [evangelical church’s] binary makes it, makes people really vulnerable I think to abuse,” and that “their identities will be subject to questioning because of what people believe around sexual abuse and gender and sexuality” (P13). Though diverse in nature, the myths and misconceptions led to a reduction in seeking support and “to a lot of doubt and shame, and implications like that” (P17).

*From the Experiences of Service Providers:* Many service providers also alluded to how police or judicial systems downplay the gravity of SGM IPV due to persisting myths and misconceptions. One female identifying service provider stated how “we are not treating these relationships as frequently as we should in the justice system as intimate partners. We’re treating them as just standard assaults. And I think then we overlook a lot of the dynamics that people are experiencing because we are filling it short without understanding the whole picture” (SP1). Another participant alluded to misconceptions of self-protection in IPV incidents, describing how “they were just trying to protect themselves during the offense, or during the abuse, and so there’s often a lot of complications with that in the sense of… the police identifying them as either a perpetrator or a victim” (SP7); this quote also highlights the importance of how IPV is measured and compared particularly in the context of bidirectional IPV. An important myth in the context of judicial systems is who is “legally recognized in the courts as a child’s guardian,” particularly when one individual is the biological parent (SP4).

In relation to shelters, one service provider stated how SGM individuals receive “a lot of judgment” in shelters “because they assume people of the community are going to make passes or advances on the people being sheltered” (SP5). This person also described how “we assume that gay men would be comfortable in a space that’s primarily like coded or built for female-identifying folks because, in general, gay men are more feminine” (SP5). Many implications arise from myths and misconceptions for SGM individuals who seek help in violence shelters for IPV, such that (1) “[those who are] assigned male-at-birth, non-binary people, I do not think anybody will take at this point,” (2) “people who are sexually diverse who, sort of, pass as straight to come to shelters and to access services, and so I think that has a huge impact to peoples’ emotional wellbeing and their sense of belonging,” and (3) “if you are an assigned female at-birth person who identifies as female, or perhaps a gender non-binary but you maybe have a more feminine name, then you can access shelters” (SP2). Others paralleled this: “many of the shelters or organizations will not take folks who identify as trans for sure” (SP6) and “you have an individual from the transgender population who identifies as female, or identifies as male, but has to be put into a female [or male] shelter” (SP7).

One service provider described how SGM individuals experiencing IPV think they are not “going to be taken seriously” and “that they were going to tell me I was making a big deal out of nothing” (SP1). This participant also described invalidation within the community due to myths and misconceptions such that social supports may say “no you are not, you just need someone to pay attention to you” (SP1). Lastly, the service provider also described how they “do not think children from intimate partner violence are getting the same level of support when that happens because it’s just ‘mom got into a fight with a friend’ versus ‘mom got into a fight with her partner’” (SP1). A service provider explained the implications of individuals believing that gender or sexuality is static, such that partners may become abusive when the other “person starts to really examine or really sit with their identity and really grapple with it, because there’s a mourning process the [partner] has to go through because the person that they were involved with may not be the [person] that they thought” (SP3). Alongside the discourse of mental health and substance use challenges, a service provider alluded to how “we do not recognize the youth that are experiencing IPV and the challenges, the unique challenges, that they are having just growing up” and that “it prevents them from stepping forward and asking for help” (SP7).

### 3.5 Theme 5: recommendations and areas for improvement

Recommendations were identified by both those who experienced IPV and service providers. These recommendations were classified as those involving professional, organizational, and systems-level changes, those targeting increased SGM representation, those improving the safety and quality of life of SGM individuals, those increasing educational awareness, and those discussing research practices. Please refer to Table 2 for a summary and comparison of recommendations aimed at addressing myths and misconceptions related to IPV from the perspectives of those experiencing IPV and those providing support.

**Table 2:** Table of recommendations for addressing IPV.

Type of recommendation	Recommendations from those experiencing IPV	Recommendations from service providers
Professional-, organizational-, and system-level changes	Improve the delivery and comprehensiveness of professional development opportunities for formal supports to target their responses specific to the needs of SGM individuals. Such recommendations for professional development include an emphasis on inclusivity and sensitivity training, the role of intersectionality (Crenshaw, 2017), and education on the heterogeneity of SGM individuals Increase inclusivity of resources and training in domestic violence settings Complete vetting processes when hiring to ensure competency in professional role and to reduce hiring of individuals with biased or discriminatory attitudes Employ SGM individuals with other intersecting identities (e.g., visible minority, disability) to increase representation among staff members and promote comfortability; decrease barriers for minority groups seeking employment to increase representation of identities Showcase qualifications and training of counselors to enable individuals experiencing IPV to make optimal decisions regarding help-seeking Increase the number of online options; provide online resources and the number of supports that can help through online platforms Increase the number of reporting outlets beyond just police	Decrease bilateral discrimination arising from oppressive policy and legislation (decrease discrimination from government bodies) Increasing exposure of SGM-related health problems (e.g., IPV) in the media Utilize a trauma-informed approach in service provision Employ SGM individuals with other intersecting identities (e.g., visible minority, disability) to increase representation among staff members and promote comfortability; decrease barriers for minority groups seeking employment to increase representation of identities Increase inter-organizational collaboration and engagement between sectors
Increase SGM representation	Emphasis on the importance of peer support groups run by SGM volunteers Pull resources and guidance from experience rather than just an outside lens Create formal support settings specifically for SGM individuals Improve the provision of services for SGM IPV in rural areas Decrease division of services based on gender identity to reduce segregation; offer opportunities to access services or organizations that are not divided by gender Have SGM champions in school environments Increase representation of SGM individuals in legal settings	Allow SGM individuals with lived experience a voice in decision making at higher systems change (e.g., where funding should be directed, how policy should be changed) Employ individuals with similar identities to increase representation among staff members and promote comfortability Create formal support settings specifically for SGM individuals Consider each respective SGM identity group when providing formal support
Improve safety and quality of life	Improve the safety of individuals experiencing IPV by providing opportunities to permanently disrupt the cycle of violence, e.g., minimize the extent to which those engaging in abusive behaviors can interact with their partners in legal settings Provide support to individuals who experience IPV without questioning their experience or requiring evidence (primarily for informal supports) Increase employment opportunities to increase quality of life and reduce the potentiality to experience adversities or engage in risky health behaviors Provide long-term support rather than short-term support which involves skill-based support to individuals experiencing IPV	Change current systems in place (e.g., policies, government bodies) to reduce marginalization Increase awareness about SGM health inequity to increase funding toward improving healthcare access for SGM individuals. Consider an intersectional approach to supporting individuals experiencing IPV
Educational awareness	Provide more educational opportunities about IPV to SGM individuals within formal support settings to prevent and address IPV Improve how elementary and high school curricula teach students about IPV, extending sex education to include gender and to diverge beyond simply a cishet discourse Educate police on stigma pertaining to SGM and racialized groups to increase inclusivity and optimal care Increase education specifically regarding consent Utilize workshops with more meaningful engagement including role play to educate service providers Increase awareness of different forms of IPV to increase help-seeking behaviors	Increase training and education to increase understanding about non cishet IPV Promote awareness in the general community to allow SGM individuals experiencing IPV to not only be cognizant of available services and resources, but to also perceive safety and inclusivity within them Educational programs for service providers should extend beyond annual and repeating educational sessions to have a foundational understanding; provide more options to increase inclusivity and accessibility in the workplace
Methodological considerations	Identify unique risk factors for IPV among each respective SGM identity group to help with prevention efforts	Improve the tracking of crime statistics pertaining to IPV among 2SLGBTQIA+ individuals

## 4 Discussion

This qualitative study sought to develop a compendium of encountered myths and misconceptions based on interviews with SGM individuals who experienced IPV and relevant service providers. Findings were organized under five main themes, including (1) myths and misconceptions about IPV more generally and its prevalence, (2) IPV myths and misconceptions in relation to gender and sexual identity, (3) societal factors influencing myths and misconceptions and IPV experiences, (4) impacts of myths and misconceptions of gender and sexual identity, and (5) recommendations and areas for improvement. Subcategories included myths and misconceptions pertaining to: the prevalence of SGM IPV, consent, the healing journey, respective sexual orientation and gender identities, and intersections with race, religion, and body image.

Overall, the unintended consequence of the histories of gender-based violence, which has mainly focused on the disproportionate experiences of violence against women, has led to further marginalization of the experiences of SGM groups (Cannon and Buttell, 2015; Gill, 2018). For instance, much of the language around IPV is centered around violence or abuse against women, which has “left behind” or underrecognized individuals who fall outside of this norm. However, it is known that SGM groups experience IPV at similar or greater rates as cishet couples (Davis and Crain, 2024), highlighting the need to increase capacity building in research, policy, and programming to include SGM groups (Nash et al., 2023).

### 4.1 Body image is a prominent factor to consider in addition to sexual orientation and gender

Body image was a pertinent factor discussed in the context of SGM IPV by both individuals experiencing IPV and service providers, intersecting uniquely with gender and sexual orientation. Fat people, that is, people of a larger body size, the word being reclaimed by fat activists, live in a world and a society through which their identities are constantly devalued and degraded, much like SGM individuals (Royce, 2020). Therefore, SGM individuals who are also fat must navigate compounding misconceptions which can influence their IPV experience. Some participants in our study described the challenges that they encountered when seeking support if they experienced IPV in a same-sex relationship and also self-identified as the bigger partner; for example, supports minimized the gravity of IPV that participants experienced since they were the same sex as their partner and also appeared less vulnerable given their weight. Even though fat individuals are more susceptible to violence (Royce, 2020), there appears to be a misconception that fat individuals are somehow stronger or harder to enact violence against. This was further confirmed in our study whereby larger-sized participants expressed shock or experienced shocked responses from others when they experienced or disclosed IPV, respectively, as the bigger partner in the relationship. However, the reality is that such relationships have a power imbalance which an abusive straight-sized partner can exploit (Royce, 2020). Specifically, shaming individuals for being fat leads to lower self-esteem, facilitating abusive partners’ capacity to coercively control their partners by convincing them that no one else will want them (Royce, 2020).

The physical embodiment of feminine versus masculine energies was also linked to internal and external perceptions of the nature of IPV. This was particularly evident for lesbian or gay relationships, such that participants believed they needed to embody certain identities or characterizations (e.g., be more feminine) for their relationship to function, fitting molds that were created from cishet understandings (Johns et al., 2012). Further, professionals were less likely to intervene when same-sex partners appeared similar in their gender (e.g., both masculine) as these types of gay or lesbian relationships diverged even further from the cishet man-on-woman or even butch versus femme same-sex IPV scenario. Findings from a scoping review paralleled these findings through vignette studies such that police consistently maintained biases and misconceptions pertaining to same-sex IPV, which attenuated the gravity of the situation and prevented adequate responses (Kurbatfinski et al., 2023). Preconceived notions of what gender entails within abusive relationships remain a direct manifestation of endorsing cishet man-on-woman narratives which must be eliminated to reduce bias and promote equitable provision of care to SGM individuals.

### 4.2 Race and religion influence perceptions of IPV among sexual and gender minority

SGM individuals who also identify as a racial minority are exposed to more myths and misconceptions pertaining to IPV due to intersecting oppressions that target their overlapping identities as a SGM individual and a racial minority, an idea supported by the minority stress model (Lick et al., 2013). Within SGM relationships, perpetuating beliefs that certain racial groups should have specific roles or exhibit certain behaviors in their intimate relationships (e.g., Asian men should be hyperfeminine) can lead to coercive behaviors which conform racial minority individuals to fit desired molds. In the context of same-sex relationships, which are already impacted by misconceptions that classify partners into certain roles, racial minority SGM individuals may feel even more boxed into a specific role. Consequently, these individuals may experience cognitive dissonance, or a misalignment between one’s behaviors and one’s beliefs or preferences; conversely, individuals may experience IPV if they resist conforming to such roles. Therefore, SGM individuals who self-identify as a racial minority must simultaneously navigate the misconceived and prescribed roles attributed to their sexual orientation, gender identity, and race, further compounding their IPV experience.

Across sectors, systemic racism continues to oppress racialized SGM individuals, resulting in myths and misconceptions that reduce or even eliminate access to varying services and resources and convolute professionals’ perceptions regarding SGM intimate partner relationships. For example, studies show that Black gay men are more likely to be arrested compared to white gay or heterosexual men (Hirschel and McCormack, 2021), which is unsurprising given that incarceration rates (a result of racism and systems founded on colonialism) remain consistently higher among persons of color (Owusu-Bempah et al., 2023). This embodiment of racist beliefs that racial minority individuals are more dangerous or aggressive than white men leads to pervasive misconceptions that undermine equitable service provision.

Tying in with patriarchal contexts, some religions maintain beliefs that female-presenting individuals should remain at home and embody “wifely duties”; abusive partners may utilize these beliefs to attenuate their partner’s engagement in society, ultimately increasing their partner’s reliance on them and decreasing the capacity for help-seeking to occur (Ross, 2014). These beliefs can also be used to rationalize the use of abusive behaviors (Ross, 2014). Individuals who do not fit the roles that their religions uphold may be shamed by their partners (Ross, 2014). Alternatively, individuals may minimize their IPV as they believe it is normal and deserved (Ross, 2014). Service providers with biased religious views may also minimize the experiences of certain individuals who act differently from their religious understanding. It is important to note that religious figures (e.g., pastors) can serve to support individuals experiencing IPV, providing an informal outlet for support that can facilitate help-seeking. Adopting religion in the discourse of IPV and related myths and misconceptions is imperative for SGM individuals as it is a powerful ideology that can further exacerbate and mold how one understands gender and intimacy.

### 4.3 Defining consent: conceptualizations differ based on gender and sexual orientation

Myths and misconceptions related to consent in SGM relationships arose in the interviews as an area of impact for SGM individuals experiencing IPV. While research indicates that SGM individuals experience sexual assault at rates equal to or higher than cishet young adult women (McKenna et al., 2021), unique and nuanced factors impact the sexual violence experiences of SGM individuals, particularly as it relates to sexual consent. Myths and misconceptions specific to SGM individuals (e.g., stereotypes based on sexual orientation) and contexts such as bathhouses, “kink” interactions, “hook-up culture,” or “top-bottom” dynamics may impact the perspectives of SGM individuals and those of formal and informal sources around sexual consent. Additionally, a culture of heteronormativity may contribute to judgment, blaming the person who experienced the violence (in some cases, self-blame) or minimizing experiences of sexual violence in SGM relationships and impact the willingness of SGM individuals experiencing sexual violence to disclose these experiences. Consent, though emphasized in the context of sexual activities, must also be extended into other contexts such as physical or emotional behaviors to ensure that partners are respecting each other’s physical and emotional capacities. Ultimately, it is important to consider the language and the terms used when teaching consent and to extend programming on consent beyond more traditional narratives to include SGM-specific dynamics.

### 4.4 Impacts of myths and misconceptions: comparisons with cishet relationships

As the experiences and perspectives of SGM individuals who experience IPV have traditionally been left out of mainstream discourse on the issue of unhealthy relationships (Cannon and Buttell, 2015), ranging from a lack of media portrayals of IPV in SGM relationships to missing SGM IPV representation in policy discussion or service design and delivery, and further compounded by socially embedded homophobia and heteronormativity, the impacts of myths and misconceptions related to SGM IPV are varied and significant. Impacts related to help-seeking outcomes predominated throughout this discourse. This may be due to misconceptions carried by formal or informal sources of support as well as SGM individuals’ first-hand experiences of these misconceptions, both having the potential for negative consequences for individuals experiencing IPV who disclose their experiences. This is further supported by a plethora of vignette studies which show decreased perception of severity when examining cases of same-sex IPV (Basow and Thompson, 2012; Poorman et al., 2003; Wasarhaley et al., 2017) or studies that demonstrate increased rates of dual arrests among same-sex gay couples (Hirschel and McCormack, 2021). Concern that police or other formal sources of help would minimize, misunderstand, or lessen the gravity of the IPV resulted in hesitance to seek help; falling outside of the cishet norm of how IPV is conceptualized, SGM IPV was deemed invalid and challenged the help-seeking experiences of SGM individuals who experience IPV. Additionally, a lack of knowledge of the existence or context of IPV in SGM relationships led to incidents of IPV being treated as assault between friends or acquaintances by formal sources of support, resulting in the nuances of IPV being unrecognized and unaddressed and, in some cases, being inaccurately treated as bidirectional IPV.

SGM IPV was also ignored by medical professionals when the abuse or violence was non-physical. This lack of adequate healthcare service provision can further compound health inequities faced by the SGM population; for example, all forms of IPV have been positively associated with anxiety, and all, except physical abuse, have been significantly associated with depression (Henry et al., 2021). Healthcare professionals must be cognizant that in addition to the toxic stress that is experienced in the presence of IPV, SGM individuals must also navigate discrimination (e.g., homophobia) throughout the lifespan, resulting in hypervigilance and a heightened stress response system which may lead to several health conditions (Lick et al., 2013); this is further compounded for SGM individuals who experience other vulnerabilities (e.g., discrimination due to race or disability). The pervasive impacts of minimizing SGM IPV experiences or neglecting to provide inclusive care are numerous and ultimately perpetuate outdated misconceptions founded on patriarchal understandings. These misconceptions, which manifest in one way or another in other public health issues (e.g., substance use disorders, HIV), must be addressed prior to planning and delivering healthcare policy and interventions to accurately include SGM individuals.

### 4.5 Recommendations directly from those with experience of IPV or who provide support

Numerous recommendations for how services and resources could be enhanced for SGM individuals who experienced IPV due to myths and misconceptions were conveyed by participants, such as: (1) strengthening professional development opportunities for formal support systems aimed at tailoring responses to SGM individuals’ needs through inclusivity and sensitivity training with specific strategies including the implementation of vetting processes during hiring to ensure competency and reduce bias; (2) increasing SGM representation among staff; (3) promoting inclusive resources and training among service providers; (4) diversifying reporting outlets for SGM individuals experiencing IPV beyond law enforcement; (5) advocating for peer support groups led by SGM volunteers, and establishing formal support settings tailored for SGM individuals; (6) developing comprehensive curricula for educational institutions and public workshops aimed at empowering SGM individuals to recognize and address IPV; and (7) identifying unique risk factors among specific SGM groups in order to counteract IPV and improve the provision of services for rural SGM individuals experiencing IPV.

Service providers working with SGM individuals who have experienced IPV also provided recommendations for the enrichment of services and resources. At an institutional level, service providers highlighted the importance of increasing media coverage of SGM-related health issues, employing trauma-informed approaches with SGM individuals who experienced IPV during service provision, considering the needs of each respective SGM identity group, and abolishing discriminatory government policies and legislation. Vital steps that were envisioned by service providers to achieve these institutional objectives included hiring SGM individuals with intersecting identities at service agencies, cultivating inter-organizational collaboration, and empowering SGM individuals with lived experience to participate in decision-making processes. Importantly, service providers also spoke about the importance of public awareness campaigns aimed at conveying the safety and inclusivity of services and resources for SGM individuals who experience IPV. Finally, service providers broadly spoke to the significance of enhancing crime statistics tracking related to IPV among SGM individuals to optimize interventions.

### 4.6 Limitations and strengths

Some gender and sexual orientations were not represented adequately in this study (e.g., asexual, intersex), limiting findings for these specific identity groups; however, the study included Two-Spirit, lesbian, gay, bisexual, trans, queer, questioning, pansexual, non-binary, and polyamorous individuals and was rich in data. Our findings could have been impacted by our biases and subjectivity; however, multiple authors reviewed the same transcripts to increase objectivity and authors met repeatedly to limit any biases from influencing the findings of this study. Further, the participants were somewhat racially and socioeconomically diverse, strengthening the impact of this study. Lastly, collaboration with community agencies that directly support SGM individuals experiencing IPV enabled more directed and meaningful preparation and clarity of the manuscript.

## 5 Conclusion

This research allowed for the development of a comprehensive compendium of myths and misconceptions that SGM individuals experiencing IPV and relevant service providers encountered. The lived experiences highlighted by participants illuminate the complex ways SGM groups navigate IPV and negotiate power and oppression both within and outside their community networks. These social expectations, both within and outside of the SGM community, may cause individuals to underreport and silence experiences of IPV. This can create unequal levels of risk due to myths and misconceptions which prevent SGM individuals from seeking help and accessing appropriate IPV programs. The power of narratives, such as those found in this research, can be used to guide future research and to design intervention and prevention programs that specifically address the needs of SGM individuals impacted by IPV, and ensure that individuals, regardless of sexual and gender identity, have access to the support and resources they need when facing IPV in their lives.

## Data Availability

The raw data supporting the conclusions of this article will be made available by the authors without undue reservation.
