# Supplementary material for: Myths and misconceptions of intimate partner violence among sexual and gender minorities: a qualitative exploration
Source: Front Sociol. 2024 Dec 20;9:1466984. doi: 10.3389/fsoc.2024.1466984 (PMC11695346; doi:10.3389/fsoc.2024.1466984)
Supplement: Supplementary file 1 [file Table_1.DOCX]

**Table A.** Consolidated Criteria for Reporting Qualitative Research (COREQ) guidelines

| **Item No** | | **Guide Questions/Description** | **Reported on Page #** |  |
| --- | --- | --- | --- | --- |
| **Domain 1: Research team and reflexivity** | | | |  |
| **Personal Characteristics** | | | |  |
| 1. Interviewer/ facilitator | | Which author/s conducted the interview or focus group? | Pg 7 |  |
| 2. Credentials | | What were the researcher’s credentials? E.g., PhD, MD | Pg 1 |  |
| 3. Occupation | | What was their occupation at the time of the study? | Pg 1 |  |
| 4. Gender | | Was the researcher male or female? | N/A |  |
| 5. Experience and training | | What experience or training did the researcher have? | Pg 7 |  |
| **Relationship with participants** | | | |  |
| 6. Relationship established | | Was a relationship established prior to study commencement? | Pg 7 |  |
| 7. Participant knowledge of the interviewer | | What did the participants know about the researcher? e.g. personal goals, reasons for doing the research? | Pg 7 |  |
| 8. Interviewer characteristics | | What characteristics were reported about the interviewer/facilitator? e.g. Bias, assumptions, reasons and interests in the research topic | N/A |  |
| **Domain 2: study design** | | |  |  |
| **Theoretical framework** | | |  |  |
| 9.Methodological orientation and Theory | What methodological orientation was stated to underpin the study? e.g. grounded theory, discourse analysis, ethnography, phenomenology, content analysis | Pgs 8 and 9 |  |  |
| **Participant selection** | | |  |  |
| 10. Sampling | How were participants selected? e.g., purposive, convenience, consecutive, snowball | Pg 7 |  |  |
| 11. Method of approach | How were participants approached? e.g., face-to-face, telephone, mail, email | Pg 7 |  |  |
| 12. Sample size | How many participants were in the study? | Pgs 10 |  |  |
| 13. Non-participation Setting | How many people refused to participate or dropped out? Reasons? | N/A |  |  |
| 14. Setting of data collection | Where was the data collected? e.g., home, clinic, workplace | Pg 7 |  |  |
| 15. Presence of nonparticipants | Was anyone else present besides the participants and researchers? | Pg 7 |  |  |
| 16. Description of sample | What are the important characteristics of the sample? e.g. demographic data, date | Pg 10 |  |  |
| **Data collection** | | |  | No |
| 17. Interview guide | Were questions, prompts, and guides provided by the authors? Was it pilot tested? | Pg 7 and Supp Mat |  |  |
| 18. Repeat interviews | Were repeat interviews carried out? If yes, how many? | N/A |  |  |
| 19. Audio/visual recording | Did the research use audio or visual recording to collect the data? | Pgs 7 and 8 |  |  |
| 20. Field notes | Were field notes made during and/or after the interview or focus group? | N/A |  |  |
| 21. Duration | What was the duration of the interviews or focus group? | Pg 8 |  |  |
| 22. Data saturation | Was data saturation discussed? | Pg 9 |  |  |
| 23. Transcripts returned | Were transcripts returned to participants for comment and/or correction? | N/A |  |  |
| **Domain 3: analysis and findings** | | |  |  |
| **Data analysis** | | |  |  |
| 24. Number of data coders | How many data coders coded the data? | Pgs 8 and 9 |  |  |
| 25. Description of the coding tree | Did the authors provide a description of the coding tree? | N/A |  |  |
| 26. Derivation of themes | Were themes identified in advance or derived from the data? | Pgs 8 and 9 |  |  |
| 27. Software | What software, if applicable, was used to manage the data? | Pg 8 |  |  |
| 28. Participant checking | Did participants provide feedback on the findings? | N/A |  |  |
| **Reporting** | | |  |  |
| 29. Quotations presented | Were participant quotations presented to illustrate the themes/findings? Was each quotation identified? e.g., participant number | Pgs 12-27 and suppl mat |  |  |
| 30. Data and findings consistent | Was there consistency between the data presented and the findings? | Pgs 12-27 and suppl mat |  |  |
| 31. Clarity of major themes | Were major themes clearly presented in the findings? | Pgs 12-27 and suppl mat |  |  |
| 32. Clarity of minor themes | Is there a description of diverse cases or a discussion of minor themes? | Pgs 12-27 and suppl mat |  |  |

**Table B.** Table of quotes supporting themes and subcategories

| **Theme** | **Quotes** |
| --- | --- |
| **Myths and Misconceptions about IPV More Generally and its Prevalence** | **General Remarks (n=13):**  “it’s not as serious as when a man hits a woman which is ridiculous because it is. But, like, if it happens, like a girl can’t do as much damage to another girl or a man can’t do as much damage to another man. Like, that’s the biggest misconception out there.” (P1)  “But it’s not spoken about and that’s a huge problem because it’s not, like, you don’t hear people talking about it. You don’t see it on tv or media or anything like that. And, I think the sensitivity training in authorities as well, they talk about male and female, male and female, man hits a woman, man hits a woman and that’s the extent of it. And so, it’s not seen. That’s the problem. It’s not seen.” (P1)  “How relationships in general are portrayed. Because like, even straight relationships, it's so normalized for there to be hatred, and, you know, generally screwed up stuff.” (P2)  “[Media] doesn't really show the intricacies and that kind of thing that show up in a queer relationship.” (P2)  “with standards of how people in a certain gender should act… I find it ridiculous that people think that women are, that women can't abuse someone.” (P3)  “domestic violence is taboo and stigmatized, that it is men against, cis-men against cis-women. People don’t see that women are capable of that, people aren’t reporting it” (P9)  “‘Oh well, a woman only faces a woman because she was sexually abused by a man and so she can no longer feel safe being with men’ and it’s not that, like, people are naturally homosexual. It’s that they were abused into being homosexual” (P13)  “she has a more gender-neutral name, it really could be interpreted as male or female. I think they didn't even pick up on that. So because she wasn't present” (P16)  “I don’t think attacking like gender identity and being like well obviously like they’re, they’re a man…well that’s obviously a man because women wouldn’t do this” (P17)  “when the perpetrator is male, or sometimes even a trans-woman because our society is trans-misogynistic, is the abuser it’s like ‘Oh yeah...’ but like lesbian intimate partner violence or like a non-binary abuser, that’s not really, it’s not seen as as legitimate… woman can’t be as abusive, or like queer couple are automatically healthier than straight ones” (P18)  **Commonality of 2SLGBTQQIA+ IPVA (n=9):**  “No, because I don’t think people even know how common it is, and in a way, that it’s kind of accepted because people don’t really view 2SLGBTQ people as like whole” (P5)  “I think that there is a lot more going on under the surface that people don’t realize, particularly when it comes to, uh, emotional abuse and that manipulation piece, I think there’s a lot more of than people realize, um, and I think that there’s probably a lot of people in the community who have been victims of, um, emotional abuse without even actually realizing that that’s the case” (P7)  “wonder if they will be believed and then more than that, I feel like , there is a, I don’t know if it’s like a stigma or a stereotype, there’s a certain expectation within the community that like we have the world by the tail we have everything in the sense, we can’t let anyone know that we’re struggling. We can’t go to the straight world and tell them that we’re, you know, experiencing the same things that they do because were not straight.” (P8)  “[IPVA] is so high, and it’s not talked about enough.” (P8)  “I think just that it’s assumed it doesn’t happen.” (P10)  “people not seeing a relationship of the same sex as, like, as valid. Often they’re seen as, like, more casual and, like, not a real relationship because I have a conservative family and I think it’s just, like, not expected that there is violence in the same sex community.” (P10)  “there’s a societal assumption that almost, like, IPV doesn’t exist in homosexual relationships.” (P13)  “I think people need to talk about it more maybe. They need to realize that it is just as prevalent in the gay community as it is in the straight community… Yeah. I think a lot people in the community already kind of know about it, and they kind of just, like, laugh at it, or they’re like “This is fine”, but it’s really not fine, and it’s, like, really not fine. Basically people need to acknowledge that it’s not ok.” (P14)  “I wouldn’t say that people who are part of the community are more prone to being abusers, but I feel like it is a problem and I feel like sometimes it can get underreported or minimized… I think, sometimes in the community they try and minimize the amount of abuse that goes on so it just looks like were welcoming, friendly, happy, loving community, and then put the spotlight on like heterosexual female and male domestic abuse and, but I think that has a lot of social implications to it.” (P17)  “And you kind of feel like queer culture is healthier because it’s not as built up in norms but that doesn’t mean that it’s necessarily not abusive. So, I think, like for me, it’s the cognitive versus emotional. Like it’s ‘Ah, what the hell is wrong with the straights?’ versus ‘Hey, I think this happens here too’, and you have to kind of remind yourself that; know this happens in our community as well.” (P19)  **Healing is not uniform (n=2):**  “Just because you’ve been through a situation of intimate partner violence doesn’t mean that you’re unable to be stable and healthy in a relationship post intimate partner violence, um, or that if you’ve, if you’ve been abused in any sense that you’re going to be the victim and that you can’t be resilient and strong and the person” (P7)  “But to be able to recognize just like that you are able to do okay after, to work hard.” (P16) |
| **Myths and Misconceptions in Relation to Gender and Sexual Identity** | **Gay (n=6):**  “I do think there’s probably a lot of things that aren’t consensual or are somewhat pressured, just in conversation with friends. Yeah, I think like top bottom dynamics can complicate it” (P4)  “also for like tops who have been sexually assaulted, I think a lot of people are like how is that even possible, I thought you would have to be like bottoming for that to happen. And also like experiencing an erection while being sexually assaulted, I think people might be like well doesn’t that mean you were into it and like you were enjoying it.” (P4)  “like the way bathhouses are regarded, it’s like well you went to this space to have sex, like what do you mean you were sexually assaulted?” (P4)”  “And maybe that the people who engage in submissive sex have like no boundaries, and, like enduring things” (P4)  “Within the gay community, I would say that the amount of sexual assaults that happens is not talked about enough and it happens far too often.” (P8)  “there’s a pretty strong reputation of, like, being, like, there being some level of, like, sexual, normalized sexual touching in the gay community that isn’t, like, like non-consensual touching but I don’t know if that counts as intimate partner violence because it’s usually, like, when people are hitting on each other, or like, there’s certain, like, normalized behaviours when a gay man hits on another gay man that isn’t normalized so much with, like, queer, non-binary communities.” (P13)  “It’s like, in a lot of ways, I don’t want to make being gay a part of my identity or like, the biggest part of my identity.” (P14)  “very open, non-monogamous, and party, and you know drugs involved, and HIV and all of that stuff still sort of prevails-ish, today sort of. So, ya, lets say, ya there’s definitely some sort of a correlation there, I can’t kind of figure out how to put it in words so. Just the lifestyle that we lived as gay men, and you know living in the home where some of it was happening, and then, you know, being at like lets say a bathhouse, right? They’re more primarily for gay men, and there’s instances of verbal and mental abuse there and ya. That’s what I got there.” (P15)  “I had a friend – his boyfriend, I think they were both pretty, they were both very stunning, and both like very good looking, and just like, very well dressed. But not quite, like super feminine, but, but still very like pretty. But then they got into a big fight. And police were also involved, but I don't think they took it seriously, because I think they were just so different.” (P16)  **Lesbian (n=9):**  “I just feel like, they sent out male cops, and they looked and, like, ah it’s a cat fight, kind of deal.” (P1)  “Because, you know, don't wear that because boys are going to, boys they're going to look at you. And then you go, okay, I wear that and a girl sexually abused me and they go, okay, I don't know what to do with that… This is a situation I haven't heard of, what am I going to tell you to do? Stop being, stop being slutty but in like a lesbian way?” (P3)  “In literally all the relationships I've had, and I've had a lot of relationships. Unfortunately. Women have been the best and the sneakiest at abusing you. And you don't realize that it's happened until they're gone.” (P3)  “a lot of it was that there’s some expectation of emotional disruption in lesbian relationships, like it’s almost within the community that people accept you to be argue-y… People will separate and get back together and separate and get back together and nothing changes. And I know there’s some of that, of course, in heterosexual relationships as well but I feel like it’s almost expected in lesbian relationships… There’s like the saying among the community that the whole, like, um, UHAUL lesbian thing where lesbians immediately move in together then like are completely inseparable and there’s always a jealous one. I don’t think that that’s, I’ve heard that previously, and I think that was true maybe in previous relationships but I don’t think it’s as universally true as people think it is.” (P7)  “either like the home type of lesbian or you’re the party lesbian and there’s not really a, that same type of expectation of straight couples if that makes sense?” (P7)  “I felt like they were kind of like oh these lesbians got into it a fist fight and now were wasting our time here when we could be dealing with real things. Like it was very, like they didn’t really even think it was that big of a deal.” (P7)  “Even with my family doctor, when I told her I was experiencing abuse within the home and I didn’t know who else to go to, I didn’t feel like it was a police matter because it hadn’t escalated enough that I was coming in with broken bones or bruises but I needed to talk to somebody. She looked at me she’s like you’re fine… No-no-no, and because it was a woman, and I-I, she’s like you guys are equal and I said no we’re not actually though like, there’s a severe bully and victim issue, or like a narcissist and, and victim issues.” (P8)  “They would look at me like well I mean you don’t have sex, like I’m not really sure why you think that would even remotely affect you. And they-they would be so dismissive as to think that between two women you can’t have sex (chuckles).” (P8)  “I think one of the biggest ones is that, like, women wouldn’t participate in any kind of violence to each other, and yeah.” (P10)  “I think people just kind of thought it’s like ‘Oh, they’re just fighting a little bit’ but it was more than that.” (P10)  “I don't know what the proper term is, but I think like for more butch women that I've met or know of, I think they're, I think a lot of it and it starts off I think like for figuring out who you are, and figuring out your orientation, you always feel like you need to fit some kind of stereotype. So like playing this role, playing like be more butch and be more tough and be more hard and just like more masculine, I think that also affects how they portray themselves, and how they treat people as well. And I think also that has to do with like, internally, like, you're very conflicted, you're like, ‘okay, so I need to, like, be this part and play this part’, but they're still trying to figure out who they are, what they are. And not everyone. I'm not saying like, every butch person is going to be like that. But I think they do tend to be more aggressive at the start with people when they're trying to figure that out.” (P16)_  “it was like a female partner and we were both like in their eyes like two women that was like, typically didn’t believe me, because like how do you get raped by a woman… I didn't really understand it which is just very like heteronormative and people are like, how can someone be raped if there's no penis involved kind of thing (laughter)” (P18)  “it's hard because I'm experiencing like, inappropriate stuff from someone again, who's female presenting, again, not really taking seriously because they're like, oh, you know, women like they're catty. Again, and it's just like, like, the way it's viewed as if like, oh, there's two women fighting. Like, two people in a romantic relationship. Who are our like, you know, in an unsafe situation, like, it's just like people like, don't fully view us yet.” (P18)  “I think one good example would be love bombing versus the joked about how lesbians get together like really quickly, like having somebody like, and I had to learn this the hard way because my ex did this, like somebody like madly in love with you like immediately and planning everything, that’s not you just u-hauling, that can be a sign of abuse.” (P19)  **Trans (n=6):**  “the idea of women abusing a man was treated with like, amusement, you know, like, like, okay, yeah, she's gonna she's gonna say that, but like, we all know, girls are crazy. She doesn't really mean it. You're just gonna have to put up with it. That's not abuse. It’s fine. Women can't abuse you.” (P3)  “Because we could be not taken seriously like being identified as a trans female now, it's harder because if you go for abuse or anything else, the police look at you as oh, you're still a man. So you know, you're the one that's being the abusive or you know, you should be able to handle it and take it.” (P6)  “I think a lot of times trans folks don’t want to report it as, you know, because then it just kind of keeps with that stereotype that a lot of trans folks are trying to fight against that you know, they’re like violent or dangerous, the same reasons why you know a lot of people are uncomfortable with having gender neutral bathrooms” (P9)  “Nobody wants to be emasculated by a woman, let alone a trans woman. So that’s, that’s one thing that I guess has kind of saved me a lot is I always figured, I get in a fight, they beat me up, it looks bad, it looks like a hate crime, it’s real bad. If I beat them up, it’s basically, when it comes down to it, you got beat up by a boy in women's clothes and then they’re really embarrassed.” (P12)  “transmen cannot be misogynistic… they can still be misogynistic and express misogynistic ideas toward their female partners for example.” (P17)  “Well, you used to be a woman, how can you hate women you know how it feels.” (P17)  “Like it’s the thing where trans-femmes, especially non-binary trans-femmes can be discriminated against in woman and non-binary spaces because they’re still flagged, even sometimes in our own community, as men which is disgusting but happens.” (P18)  **Two-Spirit/Queer/Questioning/Bisexual/Pansexual/Polyamorous/Non-Binary (n=7):**  “People, like, think that, you know, queer couples don’t experience violence, like, it’s stigmatized, again, like, I hate going back to that word because I’ve said it so many times but it is. And like, people view intimate violence or domestic violence as a thing that happens with straight couples, like male and female, and it kind of gets brushed aside and put to the wayside that it happens in queer couples.” (P1)  “media perception is not great, because a lot of queer-based media is very much so it's either, you know, these kids are gay and depressed, and one of them dies at the end, or really choose the wrong” (P2)  “I guess like community-wise too like I think like there’s a really big issue of like intimate partner violence and within like bisexual identity… the blame is sort of placed on me for, you know, even going for a male” (P5)  “I later tried to disclose my [bisexual] identity to [a counselor] and she just told me I was just wrong and confused cause I’d just gotten out of this relationship.” (P9)  “Well, what do you mean you’ve got 4 partners or 12 partners? What do you mean you fucked six guys that one night?” (P15)  ““ya, it’s like if you actually hated men you would identify as a woman and like if you don’t blah blah blah and like, uhh no you should identify as lesbian only… they said to me, it’s like oh well let me guess you have a boyfriend now or something, and I’m like again invalidating the fact, like it’s like you were never actually like bisexual, pansexual, you’re just like faking it for attention” (P17)  “there's still people who are like, okay, lesbian and gay, sure. But like, bisexual that's not real. You're just confused. Okay? No.” (P18)  “Because like as a Two Spirit person, like as a queer person, I was saying earlier, like, we're like, like trans bodies are really satisfied identify as like trans non binary, like, non gender conforming. But like, like, my body's, like, not a woman's body. Like, like, like, like I like, Yes, I have, like, female anatomy in Oregon, and stuff like that. But like, like, I'm not just a body with like, a lot of people especially being like set asides. Like, like, you're just like a body used for sex. And like, I've even had people tell me like, well, you have the right parts.” (P18) |
| **Societal Factors Influencing Myths and Misconceptions and IPVA Experiences** | **Race (n=6):**  “the hyper-sexualization of Black men I think just in terms of like Black guys always being sexual, and then well I'm that myth, can harm everyone involved in situations and conversely the feminization of Asian men.” (P4)  “also, my partner was Indigenous and had some experience within the system so I felt like if they did take it serious, they’d take it too serious. If that makes sense.” (P10)  “I still often find myself being like ostrocized. And also, like because of my like background as well like my skin color and the way I identify like, quote racially, like all of those like intersectionality things have a lot to do with like, I’m not always viewed as myself, like I'm always like viewed through the lenses of other people and their ideal if that makes sense. Yeah, so yeah, that is really challenging because I’m not able to be authentic. Like a lot of who I am like gets undermined and like, not fully seen.” (P18)  “he’s like a white man like he knew that like the police were gonna listen to him and like not me and that like he had like the advantage there” (P18)  “There's so much misinformation, right? Like, how would you expect? Like, even people within the queer community, I don't even do like to have LGBTQ plus I like it's like, people are just like, LGBT. And I'm like, oh, like you actually left out to spirit. And people are like, well, that doesn't matter. Like, and I'm, like, pretty sure you reside on stolen land, and actually does matter. And people are like, Oh, well, like, that's just like, too confusing. And it's to, like, you know, not have to, like, add more letters than and then people are gonna ask me questions after that, talk to them about it. And I'm like, exactly. And they're, like, you know, like, I'm like, wow, I must be white, like, and they're like, Well, I'm trans. And I have to deal with this stuff. And I'm like, Yeah, but… Like, we, we all have to be educated and aware of each intersectionality.” (P18)  “It’s just like it’s happened a couple times where I know like I would be accused, like my ex was like upper tier, wealthier Chinese and I know some stereotypes would say well that’s what the culture’s like… if it’s a racial thing, it’s not usually people of color who do it but other like white leftists, where they accuse you of blowing things out of proportion even though you’re genuinely hurt.” (P19)  **Religion (n=2):**  “it’s kind of complicated and tied back to, like, the evangelical church. But, like, growing up in an environment with such a strict understanding of the binary of, like, gender and sex” (P13)  “just because there’s that, like, at least within the Christian community, I don’t know if it’s society wide, that like if you’re sexually assaulted or sexually abused, that will shape your sexuality or gender in some way towards being a part of the LGBTQ+ community, and people don’t want their gender and sexuality questioned because of that.” (P13)  “within the church community that I was raised in, women are blamed for being victims of sexual violence… like the environment I was raised in around sexual abuse of women was, like, it’s your fault for tempting the man. And so, I think that definitely plays into it as well.” (P13)  “Like, my parents I trust quite a bit but like, I can’t necessarily talk to them about the oh my god, I can’t date this person because they’re, well they’re bi-gender but they do identify more with like female [inaudible 24:21]. My parents are transphobic. They’ve gotten better but the big issue would be that they’d be like ‘Well you shouldn’t date them anyway because you’re not the same religion’. So, like I wouldn’t get a lot of support. Like, my family would probably be content knowing that I don’t feel comfortable dating this person. So, that would be one thing but the actual talking about what happened with my ex, no, just some of the impacts.” (P19)  **Body Image (n=8):**  “bigger guys, if they experience sexual assault people might say like things around well you’re super tall like what do you, how did someone like overpower you.” (P4)  “a narrow idea of what sexual assault is, in terms of like someone bigger like pinning a smaller person down like below and is very clearly violent. So I don’t think other elements of control are necessarily understood” (P4)  “you can handle it because you're, you know, this, you look like a linebacker but you know, it's not all it's not always about physical it could be mental.. I was a lot heavier worked on my weight, because that was a part of my problem and everything else. But I mean, the ex was like, a skinny, very skinny and everything else. And it was like, how is she abusing you? Kind of a thing like, you clearly doubled her size.” (P6)  “I don’t visually present as a member of the community, like it’s, I’m very straight passing as they say. So like, you would never know to look at me that I have a girlfriend and have been in a monogamous lesbian relationship for years, like that’s not, something that people automatically assume. So I’m, I feel like I’m fortunate in that way, but I think that th-the fear of stigma is always there for sure.” (P7)  “‘oh, but you’re the stronger one’ can apply in homosexual relationships where it’s like ok but you two are, like, quote unquote equals. So like, there’s, it’s, I would say, like, society might perceive that as different than abuse between a male abuser and a female victim.” (P13)  “And I went to talk to them about that because I, it felt hard for me to go and talk to somebody about it because I was you know 38 and they were 18, so it was like a huge, 20 year difference. No I was 36 and they were 18 and, and being a social worker, so it was very hard, very stigmatized, and you know I’d-I’d made the wrong choice.” (P15)  “I think he was 19 at the time, the guy’s 19, how old are you. I mean is this how y’all roll in the queer community? Kind of thing. And then their follow-up on-on going to, their follow-up on my complaint it went no where” (P15)  “even if you are a bigger older person you can still be hurt and violated but there’s also that shame and guilt that goes with it” (P15)  “I think [the police] said something along the lines of like, ‘you don't seem like the regular person that I usually drive to somewhere or like see in the back of a police car” (P16)  “I guess, like the misconception thing would be just like appearance, judging by appearance. And like, yeah, I'm realizing that both partners are capable, even if they are very similar.” (P16)  “Like, they were kind of like laughing at me. And they were like, oh, like we were expecting something different. Like he thought you would like we thought you were gonna be more challenging like we thought we were gonna have to like handcuff you” (P18)  **What is IPVA (n=7):**  “It is not physical people don't really believe that.” (P2)  “Oh, just don't let them don't let them have access to your money. It was that easy. I wouldn't have been being abused, I wouldn't have awful financial patterns.” (P2)  “Because it there wasn't any, there wasn't any physical proof.” (P3)  “emotional abuse too, it’s not quite understood by a lot of people” (P10)  “It feels almost like “abuse-lit”, you know? Like, ‘Oh you weren’t, like, physically abused, like, she never hit you”; But emotional abuse is, like, almost deeper than that’” (P14)  “they weren’t ready for like the relationships we were having cause we had multiple relationships. But they were sexual, they weren’t polyamory, they weren’t romantic relationships. And they just kept getting conf-like that sort of mixed up thinking that you know there was dinner-dinners and you know here let’s go camping and go hang out and do this, and also have sex” (P15)  “like they experience a lot of drug abuse and like, physical abuse as well, but which mine was kind of like it, but it wasn't as bad.” (P16)  “why I didn't think I needed help or support was because he never like, hit me. Or, like, you never like called me names. Like, there wasn't any name calling. Or, like, any, like physical stuff, like, like, he would put his hands on me, but it wasn't like it was like, like, he was like, holding me like he wasn't, it wasn't, like, beating or anything. And like now I realized like, oh, like that's still is physical abuse, because he still like cornered me. And not let me go and like, you would like hold me down and stuff like that. But like, at the time, I was like, Oh, well, like… people who are in abusive relationships are like getting the shit beat out of them. Right? Like, I was like, that's not happening to me. So like, this isn't like it could be worse.” (P18)  “the same sex partners and stuff. I don't think like when people think of like, abuse in a relationship, it's very like back in the 50s, of beats wife. Man demands all of these things. When people specifically think of like, women, who are like predators, they always think of, like, schoolteachers sleeps with underage students, you know? And so, if, if it's not like one of those two things, then you're not experiencing.” (P18)  **Social Constructs (n=10):**  “the stigma of like, you know, I presented this, like, happy relationship and we seemed happy. And to have friends be like ‘there’s no way, like, she’s too nice’” (P1)  “You know, there's the whole idea of why didn’t you try and get out sooner why? Why did you stay all that kind of thing. Like it's, there's so much nuance and fear when you're being abused, because you can't predict your partner's next move. And if you try to then they’re going get pissed at you. And it's very easy to forget that. Sometimes people are really, really good at manipulating others. And it is an awful skill. But it's a skill that they have. And it means that most of the time until it's too late, you're not going to realize that you're being abused.” (P2)  “it's just how he is, he can't change it.” (P2)  “you know like that’s just how she is and that’s just who she is, or you just have to adapt your expectations a little bit” (P7)  “I was very like proactive about doing some of the things to advocate for myself and when I did that it was almost like the shelter was like oh, well you’re really good then” (P8)  “Especially if the person looks like they’re kind or they’re generous or they’re, you know, really a good individual overall or they put on a good front. It’s hard as the victim to feel will I, will I be believed or will I trusted that I’m telling the truth or telling how it happened.” (P8)  “It’s kind of, like, a cloak and dagger when it comes to trying to divulge issues, but-but were humans, we, everybody has issues, it’s not specific just to-to us or to our community that we have issues. The straight people have issues too but it’s almost like a faux pas that you have to go and report I don’t know sexual assault or verbal abuse, mental abuse, any kind of abuse or violence to the straight community. Because it-we [mumbles] are so scared that it’s skewing or misrepresenting the view that the straight community has of us or giving them ammo of what they think of us when we’re just all humans.” (P8)  “I ever told told me that couldn’t be true because they knew this person and thought that he was well like I a great person.” (P9)  “I don’t put myself in situations like that anymore.” (P12)  “it feels comfortable almost being with people that aren’t good to you, it’s like the opposite of what we’d think. Like, if you’ve been burned before, maybe you’d like stop doing that, but it’s actually very comfortable for me to be in a relationship where the other person is, like, using me financially or, like, putting me down, or has some sort of a mental trauma, and I end up in more relationships like that where I pick up people in my life who are, like, emotionally needy” (P14)  “I’ve told like friends or family, and usually the responses, sometimes they’re varied, but a lot of the time s it’s you shouldn’t have even been in a relationship like that anyway, there’s obviously something, like there’s obviously something wrong with them (blah blah blah)” (P17)  “it’s kind of your fault you should’ve known better hurts” (P17)  “Oh, like that poor girl has a problem. And thank goodness, this guy's here to help her kindness, making himself the hero.” (P18)  “it's not like one of those things that you regularly see in the media.” (P18)  “Well, why didn't you just leave? Or like, why didn't you just say now? Why am I my saying something now, like, you know, and it's like, everyone processes trauma differently. Like, you know, there's just like, not one answer when people are like, expect like, one storyline. And if your story doesn't follow that storyline, then it's not valid. Does that answer the question?” (P18)  “Sometimes it’s awful hard to see.” (P19)  **Consent (n=5):**  “It took me awhile because I was like consenting to aspects of it but not all of it… people struggle to understand the sexual violence if it’s not like a back alley, pinned against your will situation; like, when there’s a level of consent but not consent to everything. Or, when there’s not like a clear ‘no’. So, I guess just like general perceptions about consent have limited how much I want to talk about it.” (P4)  “I still think like power or coercion or non-physical ways of like coercing someone to do something that they’re not completely ok with, I think that’s not really recognized.” (P4)  “A lot of people have had sexual experiences that are like in the grey areas of consent and especially in hook-up culture” (P4)  “I came out of an Evangelical background where, like, we were taught that women are, like, sexual objects and don’t, like, require consent, in the way that I now understand consent.” (P13)  “Would that, like, the fact that I stayed in that relationship far past the first incident of abuse would mean that I was somehow, not necessarily responsible, but that like, by a failure to leave that relationship, that would lessen his guilt. So, like, almost a mentality of, like, why pursue it if, like, I, if by not leaving I was almost consenting to it.” (P13)  “there’s more, some sexual stuff, like me just giving in cause they won’t stop” (P15)  “So we had to go away but that resulted in going to a [bathhouse] that, you know, I wasn’t very familiar with, you know, had different policies and plays, but then the individual that I did meet, ya, it was kind of tough, because, it was part of a play that we were having, but then the boundaries were sort of crossed when we were there. Abusively crossed.” (P15)” |
| **Impacts of Myths and Misconceptions of Gender and Sexual Identity** | “It results in fear for queer couples to speak out about it, to go to authorities. And then, when they do go to authorities like the police, then it’s not taken seriously by the police; as seriously as it would be, if like say my husband hit me, if I had a husband.” (P1)  “[Due to downplay of my experience], I had to go to court and obtain an emergency protection order to have the police, like, remove her.” (P1)  “when you’re not taught anything else, you expect that to be how you’re treated. And so when you’re treated that way, you don’t find anything wrong.” (P2)  “kids don’t learn about healthy relationships. And when you’re still figuring out who you are on a very fundamental level that causes a lot of problems and it can cause a lot of projecting and a lot of very unhealthy behaviors to end up on the other person. And based on what people are seeing media, they, they people will try and base it off of that and, you know, it’s horrifically inaccurate, and it’s something that they literally don’t know. And also because kids tend to be abused for being queer. It means that the cycle of abuse just continues.” (P2)  “It makes people so much less likely to come forward and tell their stories. Because it just feels like well, why would I tell someone I have, I went through this, they’re not going to believe me, they’re not going to understand that. It’s something that deeply impacted me, they’re not going to understand that it’s something that has profound effects and like, lasting effect at that.” (P2)  “There’s, like if people don’t see physical evidence, they don’t believe you.” (P2)  “And definitely, when I disclosed to some people that I had been abused by a girl, I was met with a lot of incredulous looks, especially as a trans man. Because then it’s not girl on girl abuse, it’s girl on boy abuse and that’s even, that’s even weirder.” (P3)  “I really do, I think that it’s more of I think it’s more dangerous than it would be in in hetero, in the cis-het communities because we’ve all seen abuse in straight couples on screen. Like, we all know what it looks like. But when you are when you are young, super inexperienced, because you’re gay, you haven’t been dating a lot. You’re not going to have enough exposure to recognize abuse. When you first see it, you’re not going to recognize abuse until it’s happened to you. And someone has straight up said to you, hey, that was someone abusing you.” (P3)  “I think that places people in a, I think that it puts vulnerable people in a very hard place to be, because you are experiencing so much pain, but people are laughing at it.” (P3)  “I think that level of like how society might perceive [bathhouses], including other gay friends, or some gay friends, has an impact on like my willingness to talk about it and seek support.” (P4)  “I think like violence and consent as it relates to kink is something that probably a lot of practitioners aren’t well versed in and would probably say like stigmatizing things. So, that probably like limits the number of people who experience sexual assault within the sort of like kink dynamic , it probably discourages them from wanting to report it or get help.” (P4)  “[It is] emasculating for some people depending on their gender identity to come forward with experiences of sexual violence” (P4)  “So, they, they just don’t really think about it. So, I don’t think it’s taken seriously.” (P5)  “So harshly social stigmas and partially having to stay quiet about it because there’s really not any help out there… We shut ourselves out like, we try not we don’t trust as much, many people and it just makes life harder, more difficult in general. Because we don’t trust people or, you know, we’re we’re always thinking that somebody’s got an ulterior motive and that sort of stuff.” (P6)  “You know, nobody wants things for. Nobody does things just out of the goodness of their heart anymore. It seems like are you gonna take me and break me over the coals?” (P6)  “I think that-that, [the UHAUL myth] can be detrimental because people, who have a good fit together, um, might delay merging their lives based on that or, it might have the opposite effect, it might be that like people assume that they have to move in together right away because of that, or it’s like, that’s really the only thing that I can think of.” (P7)  “it was just kind of like well this thing that happened yup but look she’s fine now so it’s fine, and they kind of just left it at that. And I was- I was asked if I wanted to press charges, but at that point I felt already like I was taking away from their time and that I was making a big deal out of nothing” (P7)  “They, I don’t think see it as intimate partner violence, I think they just thought it was a disagreement and a physical altercation between two people. Like it wasn’t treated as if there were any indications of it being a long term abusive situation” (P7)  “Ya, so, it was very like dismissive of my doctor. My doctor was very much like oh no that’s not reality you’re not experiencing that… Like there’s a big problem and she refused to acknowledge it because we’re both female.” (P8)  “the cops aren’t going to do anything because it’s female to female violence, and the chances are she’s probably right. When in reality this person was probably was close to being dead. So it’s, it’s stuff like that to think that, the cops won’t do anything because it’s female to female violence you know, that’s not fair that’s actually not right. And it’s, it’s things like that that would cause reporting not to happen or you know cause a divide so to speak between straight and even allies and the LGBT community” (P8)  “Like, when you kind of have the idea that people aren’t going to believe you, it makes you really gaslight yourself and, like, tell yourself, like, maybe you’re wrong or you’re mistaken. And if you feel that way, like, you feel stupid for, like you can’t come forward and say anything [about lesbian violence].” (P10)  “It would definitely make you, like, less likely to report or seek out help and, like, assuming that people aren’t or, like, feeling that people aren’t going to take it seriously or believe you.” (P10)  “When you don’t fit into [the evangelical church’s] binary, makes it, makes people really vulnerable I think to abuse” (P13) “And so, I never disclosed to people who I know are from a Christian background that I was, or like, that I was ever, had experienced sexual assault because I know the, like, stigma that they were raised with around that and I, like, don’t want to perpetuate that because I was, like, identified with that before this relationship… There’s no question that a large part of, like, what made me vulnerable and also not able to seek support was I attended a church that continues to be pro-conversion therapy,” (P13)  “the situation in my own life of women [break in audio] as victims are afraid that their identities will be subject to questioning because of what people believe around sexual abuse and gender and sexuality.” (P13)  “that not having faith in what supports are there or public supports, be it healthcare, justice system, education system, to reach out. One, cause of that shame, two misconceptions, history, age, age-epidemic, all sorts of things that that come forward now, it’s just, ya kind of the key things for me.” (P15)  “they weren’t sure how to act after, they were just like, hey, this person came to us, this is what happened, so this is what we’re going to do, but not a whole lot of assessing a situation, so looking at the bigger picture [in regard to lesbian relationships].” (P16)  “He went to the hospital with like, swelling on his face, and like huge bruises on his body, and said, ‘My boyfriend hit me. And he tried to choke me.’ And the nurses and doctors didn’t take it seriously. And once again, I don’t know what the situation was like, maybe they were really busy. But I think they did take it seriously when his partner, his boyfriend – he ended up hanging himself, so this is like after the court, after they had a trial. So they actually had a trial. I didn’t. So they had a time dedicated to like, go through all the specifics, like there were cameras and security camera footage when they were outside the house when this happened.” (P16)  “Am I really a victim? Can this really be happening to me? And then overall, I think it leads to a lot of doubt and shame, and implications like that.” (P17)  “I believe that being in a LGBTQ relationship made it a little more difficult for people to kind of take it seriously, right?” (P17)  “And I feel like misconceptions like that can land in both non-LGBTQ communities and within them, so ya, so those myths stop, like are barriers to understanding the way that the intersections of violence means different identity” (P17)  “Authorities like police officers if you are to go and like get a restraining order or a police report or anything like that, I don’t they will probably take it seriously” (P17)  “Like, I don’t like or even like, was like, not like, I want to go to the police about it either. Because I haven’t had to deal with and like, even like, I don’t even think police officer would even really even get, like, me or my body or my experience either.” (P18)  “People may stay in those relationships longer or they may assume it’s love” (P19) |
| **Service Provider Perspectives** | *Myths and Misconceptions Identified*  “We had one situation… One person said it was ‘just an assault, it was just a couple of friends’, and the other one said, ‘Uh, no, you’re living here, we’ve been intimate, this is not just an assault, this is something totally different.’ But the definition that the two people had was not the same even, so we have to get to the point where we are asking and allowing people to give an honest answer and then adjusting our behavior to match what their answers are.” (SP1)  “That if there’s [parental] abuse happening within [SGM] relationships it’s not being treated as domestic violence within the schools.” (SP1)  “we’re not treating these relationships as frequently as we should in the justice system as intimate partners, we’re treating them as just standard assaults. And I think then we overlook a lot of the dynamics that people are experiencing because we are filling (can’t hear) without understanding the whole picture.” (SP1)  “Assigned male at birth, non-binary people, I don’t think anybody will take at this point. We are making those changes now. And if you are an assigned-male at birth in a same-sex relationship with another male, I don’t think anyone will take you unless you can manage to get that one male bed in Strathmore.” (SP2)  “people who are sexually diverse who, sort of, pass as straight to come to shelter and to access service and so I think that has a huge impact to peoples’ emotional wellbeing and their sense of belonging to feel like they have to compromise themselves to be able to get support.” (SP2)  “if you are an assigned female at birth person who identifies as female, or perhaps a gender non-binary but you maybe have a more feminine name, then you can access shelter. I don't, and you can access, and if you're in a same sex relationship, that's fine almost across the board I think now. I wouldn't even say that was true five years ago, so I feel like gains are slowly, slowly being made.” (SP2)  “there's not a lot of beds for male identifying people. And so, or I would even suggest trans-women who are born male at birth,” (SP2)  “Like, we tend to assume that everybody is knowing from the time that they're children, and therefore, and we also tend to assume that gender and sexuality are static, and that an identity is formed, and then it never shifts. And that's not the case.” (SP3)  “And I think a lot of that kind of cultural competency information around you know, understanding that just because on the outside the person does not look like they're part of the community does not mean that they are not part of the community because there's a lot of there's a lot of identities under the umbrella of 2SLGBTQ plus that are often really overlooked that can be greatly impacted in this way.” (SP3)  “it can get very complicated especially if, depending on how you're legally, like, are you married or are you not? Are you legally recognized in the courts as a child’s guardian or are you not? Because it’s very, if you're being abused and the partner is the biological child, who’s abusing you is the biological child's parent, they can hold that over you too and be like “You have no access to the child if you leave me. I don't care that you’ve raised them for how long. You’ll have to go to court.” (SP4)  “I think with parenting, like I said, it can get very complicated especially if, depending on how you’re legally, like, are you married or are you not? Are you legally recognized in the courts as a child’s guardian or are you not?” (SP4)  “similar to people who experience these forms of abuse, they also make the claim that ‘I wish they would just hit me’ or things like that because then ‘the people that were closest to me would actually understand that these things are happening to me and the impact that they are having on me.’” (SP5)  “So, I think there’s just a lot of judgement that comes with that. Because they assume people of the community are going to make passes or advances on the people being sheltered.” (SP5)  “we assume that gay men would be comfortable in a space that’s primarily like coded or built for female-identifying folks because in general gay men are more feminine.” (SP5)  “But one of the things to consider and I mentioned it before is that there’s no 2SLGBTQ+-specific shelter and the number of beds that are available to say, male-identified folks, are very limited in the province of Alberta, and many shelters will not accept people of the community in hopes of keeping current residents safe… Which is I think really inappropriate and not true” (SP5)  “Many of the other shelters or organizations won't take folks who identify as trans for sure.” (SP6)  “I think when I look at that spectrum – I shouldn’t say spectrum. When I look at that community, you know, I notice people are reaching out to our organization, I think there is still a fear that it’s a female-only organization, or female-identifying only organization, and that’s the risk that we run, I have female-only staff here, or identifying as female staff here.” (SP6)  “challenges in just shelter space and again you have an individual from the transgender population who identifies as female, or identifies as male, but has to be put into a female [or male] shelter.” (SP7)  “When it comes to their addiction or their mental health, because that is seen as the presenting problem, and they’re not knowing that the IPV may be precipitating those experiences, like it may be causing more harm around their mental health or the person may be actually encouraging them or making them – not encouraging them, sorry that’s the wrong word. Making them use substances in order to you know, support their own addictions.” (SP7)  “when I think about, especially people who identify as being female, when they are mandated to our IPV group by the court, often they are coming forward as a confusion to the – they’re coming forward in a way that they’ve been charged with a family violence offense and have been found guilty, that’s why they’re coming to our program, but there’s been a lot of discussion regarding that they were just trying to protect themselves during the offense, or during the abuse, and so there’s often a lot of complications with that in the sense of the you know, the police identifying them as either a perpetrator or a victim” (SP7)  “when we talk about the mainstream narrative of domestic violence there can be this idea of you know physically violence or this picture of a man who is physically violent to a woman or emotionally abusive to a woman, but I think it does change the conversations that are happening in the 2SLGBTQ+ community” (SP8)  “and saying ‘Well shouldn’t it be a fair fight’, um, ‘Can you not defend yourself against your partner?’” (SP8)  “Are you not able to do XYZ because they are about the same size as you?’ almost as if, right, there's such thing as a fair fight when it comes to intimate partner violence” (SP8)  “that there can be aspects of intimate partner violence that exist within those those dynamics, so there are considerations that we want to take because of some of these specific stigmas that may be had around sex work when it's choice-based as well as recognizing that that's very different between choice-based sex work and someone experiencing sex trafficking” (SP8)  *Impacts of Myths and Misconceptions*  “what I hear from victims that we work with, is ‘I didn’t think I was going to be taken seriously so I didn’t come forward.’ ‘I didn’t think people were going to be actually wanting to help me, that they were going to tell me I was making a big deal out of nothing.’ So, I feel like there was a lack of trust in the bigger system to believe that that help would show up and do what they needed at the time. So they waited until there was no alternative but to ask for help.” (SP1)  “I don’t know that the average 20-year-old person that identifies as gay is going to have that same confidence that their safety and their protection is going to be put first. Or more so our Trans community – I think that’s where we’re really struggling to see that sense of trust, in being able to come forward and be treated with respect and dignity. And though I feel that would be by the vast majority of the officers that we have, there’s still that sense of fear and sense of anticipation that they’re going to be mistreated.” (SP1)  “Does that make sense? So it undermines their authentic experience by them saying ‘no, I am part of this community.’ And they say, ‘Oh, no you’re not, you just need someone to pay attention to you.’” (SP1)  “we write off their opinions and we write off their experiences based on it’s a different need that you’re meeting here.” (SP1)  “So, I don’t think children from intimate partner violence are getting the same level of support when that happens because it’s just ‘mom got into a fight with a friend’ versus ‘mom got into a fight with her partner.’” (SP1)  “I don’t think our funders have an understanding about 2SLGBTQ+ folks and sadly non-profits are driven by the funding that we’re given and, you know, we do what we’re told to do with the money that we’re given for the most part. And so, because 2SLGBTQ+ folks have been left out of, sort of, that step in the government or other funders demanding that we work with folks, like, they’ve been largely ignored” (SP2)  And I think that's one of the things that can trigger intimate partner violence is when that person starts to really examine or really sit with their identity and really grapple with it, right, because there's a mourning process the other person has to go through. Because the person that they were involved with may not be the first one that they thought, right, you know what I'm saying there. So I think we just assume that people don't do that process later in life, or more than one time. Right? (SP3)  “So and I know that we did get clients come through that were part of the community. But nobody's, people aren't trained to think about how to basically unpack their biases when dealing with those people, then mistakes are going to be made and like I give people the benefit of the doubt. I don't think most people are, like, maliciously trying to, but if you don't know what could be harmful, then you're not going to be careful about how you're approaching. Right?” (SP3)  “I think there’s a lot of people who won’t seek help, because they think that they’re not going to be taken seriously, or because they don’t have the capacity to go during those hours or whatever, right?” (SP3)  “So I think a lot of times we don’t recognize the youth that are experiencing IPV and the challenges, the unique challenges, that they’re having just growing up and just the developmental stages that they’re approaching, too.” (SP7)  “I think it prevents them from stepping forward and asking for help.” (SP7)  “Those conversations have an impact, so that can actually play into the timing of when someone wants to share their story of intimate partner violence” (SP8) |
| **Recommendations** | *Recommendations from Individuals who Experienced IPVA*  “Talking about it, like, including information about healthy relationships, in school curriculum during sex ed. Including stuff about abuse is never most of the time, not just physical, abuse has many forms. Abuse is a very complex thing, and not a reflection of who you are. We need to teach kids about abuse if we have any hope of keeping them from being abused.” (P2)  “And like teaching about things like love bombing, and the cycle of abuse. Things like that so that people can be like, hey, you just did something that’s really screwed up. Can you talk about that?, and like, developing communication skills is also a huge thing. Because we don’t know how to talk to each other, in general. So talking to each other about abuse is off the table.” (P2)  “vetting process for social work needs to be more, more selective. They need to look into these people’s lives and make sure that these are people who genuinely care about the victims… I told the I told the social worker that my, my father was sexually harassing me. And I explained the behavior to her and she was like, Oh, I do that sometimes to my kids to it’s fine. It’s a joke. And I was like, okay, but it’s not a joke, though. That you giving that example? Literally does absolutely nothing. Like I just disclosed sexual harassment to you. And you’re saying it’s fine.” (P3)  “I think that we need to hire people who have these experiences people who are racialized people who are LGBT, people who are disabled, people who have been abused, because who else is going to know what this feels like? If not the people who did go through it” (P3)  “queer people who experience sexual assault to have queer therapists and queer counsellors so having that available. I guess like having their qualifications listed, in terms of what training they’ve received… Yeah, so I suppose my recommendations are like training and showcasing that training so it’s clear who has it when people want to seek support.” (P4)  “domestic violence resources to like be more inclusive and get some sort of training, or to be like educated on the 2SLGBTQ+ community” (P5)  “bisexual people in the 2SLGBTQ community, and same with trans individuals, I think that we need to, as a collective, look into why two groups specifically within the 2SLGBTQ+ community experience the highest levels of intimate partner violence, and how we can be more supportive for those because I don’t think most people can even comprehend how many 2SLGBTQ people have experienced this, especially bi and trans people.” (P5)  “the biggest thing for anyone who interacts with any member of the community is to have an opportunity to discuss their questions, concerns, fears, or misunderstandings with a member of the community themselves. So someone who’s comfortable representing their population or who is comfortable speaking to them of the struggles of their population and not just some, like, written out, put together, media plan that’s just like here sit through this slideshow here’s what we will teach you because I feel like it’s really blanket… I think the biggest thing is just making sure ‘resources are pulled from experience rather than just from, um, like an outside lens who doesn’t understand it as fully.” (P7)  “I feel like for the general policing population I think there’s still a lot of stigma there as there is with the Indigenous population, I think it’s kind of a, a systemic problem that still needs to be addressed.” (P7)  “She literally just said I don’t need to see [evidence of the abuse], I just need to know that you’re safe and that was as much that she needed to know to be able to help.” (P8)  “I just wish that there was a safe haven for adults that is like the Alex. There was the community wide resource center, for a long time, and that was LGBT-geared,I think that they probably had for the LGBT, they had not safehouses but they had like connections that are LGBT safe within the City of Calgary that you could be suggested to go to. But I don’t think that the community wide resource center is up and running anymore I think it closed. Like maybe 5 years or 6 years ago.” (P8)  “I think that the biggest one is to have more kt BIPOC folks sitting in the helping world, I think having more, specifically more trans folks I think, a lot of the time it’s really important for people to have people who also are kind of similar shoes to them, like to that peer-to-peer support even if they aren’t disclosing, but it’s just a common understanding that people don’t understand” (P9)  “if you are trans, gender fluid a lot of times, you know, they don’t really, it’s hard to access shelters from what I’ve heard and then specifically with kids, there’s not a lot of male beds for these folks but then also it has the challenge of people experiencing transphobia and homophobia when they are accessing these shelters, so, I think having a space where folks can go and it’s not specifically segregated by gender which I know is hard sometimes specifically for folks like, a cis-woman who has been abused [unsure 24:04] by cis-men, they don’t, they don’t want to be around men, they might not want feel comfortable or traumatized by maybe a trans female who presents more masculine, but I think having that option for folks is really important because then people” (P9)  “if there were like queer specific resources, like, for that. Like, if there are, I’m just not aware of them. Like, if there are, more awareness. And, for me, even though it was, like, a relationship with a straight man, I always feel more comfortable going to queer spaces because I know they’re more, like, accepting and inclusive and I just feel more cared for by queer folks. So, yeah, just like more awareness and more services. Like, even if there was, like, a queer specific, like, text line or number that would be cool.” (P10)  “If I would have been able to get kind of work or whatever. I would have never ended up in trouble with police. I would have never ended up, like, in drugs or prostitution if I, that would have changed a lot. Like, that was the big things, that’s what kind of made everything different. My life could be a total different course.” (P12)  “education around gender and sexuality and consent in the school system. I think, like, I at that point had never been taught about consent, even though I was at that point in the public school system, I was home schooled originally, and I think had I been taught a better understanding, both of like what consent is and that both parties need to consent for it to be sex, and what like coercion is, and that, like, consent with the threat of violence is vitiated consent, like, those types of things, if that was taught in sex ed, as almost like a preventative measure.” (P13)  “in terms of like a supportive measure, at the time, like, I don’t know if there were even really any LGBTQ+ specific resources at my school beyond, I think there was, like, a GSA” (P13)  “So, my current counsellor actually worked in, like, previously worked with abusive, she’s worked with people who have been abused before and have been through trauma like that, so it’s very helpful to, like, have that perspective.” (P13)  “I think I think they're already doing it, because I do have friends whose spouses are in the police force, but I think like just workshops to be more aware. Sometimes, like I know for any kind of work, sometimes it's just like, oh, this is a mandatory training or like, okay I have to do this, so I don't actually know what's going through. But I know like, even when you do those workshops, that if someone is listening, then maybe, maybe someone will pick it up and kind of like internalize it and process it. So, hopefully like more meaningful workshops, where it's not just like, b.s. kind of workshop, there’s a better word, but more like, actually meaningful. Or maybe like to make it more real, just like real cases, just like this is what happened. And like role playing, like, what could you have done in that situation? Instead of like delivering information and statistics, which like, statistics do help, but I think it's like, the real stuff where this has happened here, what could we have done differently?” (P16)  “If there were online resources where I could, like I was not able to physically go out and talk to somebody and about like hey, is this something like I should, cause every time I would look up and it could be different now, I’m not sure, every time I would look up like hey should I, do I have to report this? Should I report this? Stuff like that. It was always like go to your local police station and talk to them. I didn’t have that opportunity and I also feel like that sometimes it would be intimidating to go into and tell a police officer like ya, we were in like a, cause I understand there is a lot of bias there, so I think like online resources or being able to like talk to someone online about it and see what options are going forward would be helpful, so more online options I guess.” (P17)  “And probably, again, like, you know, they probably have so many barrier like, even I know for myself, like, I'm gonna deal with a lot when I do start looking for work and like as a person of color and things like that. And like, there's a lot of barriers for them working in the field, and then trying to find them even barriers to do the right.” (P18)  “Yeah, I think, again, just like having people from like, all walks of life is always key. As we know, representation matters, like people need to be able to feel safe and understood and heard in these situations like primarily, before they can even move forward… Yeah, just like, cultural sensitivity. Understanding where people are coming from and meeting them where they're at.” (P18)  “And just like being able to, like offer, like, long term solutions and stuff, like I felt like a lot of stuff that was opportunity was just like, very, like short term. very temporary solutions. That weren't very, like skill based either.” (P18)  “And I was in a straight passing relationship so it wasn’t even like the queer resources didn’t exist. Like, I went to the queer resource centre. I worked there for a time. It’s just, I think something like, a lot of times intimate partner violence has like a certain image, and you might be able to go ‘Oh my god. That situation sucked ass’ but you don’t quite realize what the whole situation was. Like, you don’t necessarily think you were abused because you were never hit, or no one called you like a jackass or like they didn’t even insult you. And I think what would be helpful for well everyone, but queer people too, would be to I guess have a little less nuance. Like obviously if an organization needs that for their funding, absolutely, but basically make it more open. Like, you can go even, or like quiet spaces where you’re like ‘Oh, I don’t want to take away their emotional labour because I didn’t quite go through the thing’, you still, like there’s still a place for them to sit and talk, and maybe find out that they do qualify and what to do about it” (P19)  *Service Provider Recommendations*  “And we are not tracking this type of violence as much as we are a blanket across domestic violence, or intimate partner violence – it’s just one lump answer for across the province and I think we need to start breaking it out and looking more specifically at ‘What are the triggers? What are the outcomes? What is the lethality of this type of violence, so that we can start seeing what our solutions can then be?’ because it’s only once we start really looking at those answers that we can start figuring out how to help people better. So I’m making generalizations based on the few instances we’ve experienced here, but there are no statistics that are province-wide that are clear enough, I think, on what type of experience people are having when encountering IPV within this community and the law.” (SP1)  “So, yeah, so it’s like we changed the rules and gave folks more rights but we didn’t actually change any of the systems in place that continue to marginalize them, so I think there continue to be significant barriers for people who are seeking resources who are part of the 2SLGBTQ+ community.” (SP2)  “I mean I think we need training across the board. I think we need some understanding about the, sort of, binary relationship we have about, or the binary understanding we have about intimate partner relationships, and we need to look at other ways of understanding violence in relationships in general and then how we’ve chosen as a sector to respond to that crisis. Yeah, and then to make space. I think we also have networks or groups within the sector who are turf-y which causes problems across the board.” (SP2)  “I don’t think our funders have an understanding about 2SLGBTQ+ folks and sadly non-profits are driven by the funding that we’re given and, you know, we do what we’re told to do with the money that we’re given for the most part. And so, because 2SLGBTQ+ folks have been left out of, sort of, that step in the government or other funders demanding that we work with folks, like, they've been largely ignored. And so, I think more awareness within the sector to create more services.” (SP2)  “I think, and talking to 2SLGBTQ+ folks who have experienced violence to see what they want. Do they, you know, does it make sense for organizations like mine to provide service? Maybe provide specific counselors or people who have experience in those communities to be able to work with those communities. Does it make more sense for us to do the work that we do with everybody including 2SLGBTQ+ folks without it having to be, you know, specific? Do 2SLGBTQ+ folks want their own organizations that are specific to them? Like, and I think that there’s a way that we can work, like, with those communities and people who have experienced violence to be able to create something that works both for the people that are providing the service and the funders that are funding that service and the people who require the service because that, none of that has happened. And so, we have this seemingly large group of people that aren’t being formally serviced and, I mean, maybe that’s okay but I don’t think it is.” (SP2)  “You also have a lot of distress, or you can have based on the ways in which government and police services are complicit in a lot of the violence worldwide towards the LGBTQ plus, based on those histories, you may also have kind of not feeling like the services that you would need to access are safe.” (SP3)  “I would recommend that some of the more formal avenues of support, find ways to be less formal in their approach, if that makes sense. And I don't know how they would kind of be able to do it. But like I think. I do think that the Calgary police services are doing a good thing by having that diversity liaison officer who does actually do some pretty good outreach as well. But I think there’s still that like uniform peace and all of that, that I think is still going to turn people away from seeking help that might need it, even though the other thing too, for me. And this is just because of the way that my brain works, which is very high level systems focused is is that the the IPV piece, which is what we're talking about is very important. But I think it's also important to think about family violence more broadly, particularly for the 2SLGBTQ community, and for the newcomer and refugee experience. Because from the newcomer perspective, there are a lot of people even second, like second generation newcomers who are living in multigenerational households, where they could be experiencing a lot of abuse is just not intimate partner. Right. So there could be huge experiences of violence along family lines and no avenue out or no clear avenue out because, because we tend to in the sector, we tend to really like microfocus resources right. So that's kind of the only other piece that I would kind of bring up is that I think that coercive control piece is happening within families in other dynamics and often like with adult children, so again, you've got that kind of gap for adults that aren't necessarily don't have an avenue of exit and we see that in particular with our, with like, newcomers, second generation are starting to notice that a lot because they're still possibly living at home and they've got all of those cultural kind of expectations that do a lot of harm.” (SP3)  “second stage shelters for women escaping IPV and no, like there need to be more targeted community living, like an apartment building that is, you know, all 2SLGBTQ+, that has, kind of, you know, supports all within the building and targeted counselors for their specific needs and stuff like that” (SP4)  “you can't just lump them in with everybody else and hope for the best. Like, there are specific things that they need and there are specific barriers that the current programs put up for the community that need to be addressed, taken down or targeted differently.” (SP4)  “I think that as a community or communities, not to lump them all into one community” (SP5)  “Again, it’s that representation piece, so if we’re seeing it more in the mainstream media then it kinda normalizes for people who aren’t in the mainstream media that it’s not okay and that there are resources available to you and reaching out for help is a normal and totally acceptable thing to do.” (SP5)  “normalize that these are just people like everyone else, that deserve safety in their lives. That deserve to be loved and accepted and to have access to services.” (SP6)  “And part of that again I think is the education for service providers and I know that so many of our services are going to – even here, you go to almost like, you go to Canadian Mental Health and they assess what type of counselling services you should be in, then they release the money to the counselling service, but you’ve already gone now to tell your story possibly up to three times before you actually get the counselling done. And even though it is to streamline the approach and make it easier for people to access, I don’t know if it really is easier for people to access at the end of the day. And I don’t know if we’re causing more damage doing that. There may be more investment that needs to happen where we’re looking at core sets of standards that happen with service providers around our knowledge of these different systems and how we can make these warm contacts to try and you know, alleviate those stresses for the people that are coming in our doors.” (SP6)  “So I think the biggest one is the lack of education and – not just awareness, but the lack of education and embedding this within both from the high level policies to the front line action. That we aren’t doing enough in our trauma-informed practices. If there are trauma-informed practices, to actually understand the unique circumstances of the 2SLGBTQ+ community and that needs to be foundational work that’s done at every agency. There really shouldn’t, especially around human rights, there really shouldn’t be an agency that isn’t accepting of an not willing to work with anyone within that community.” (SP6)  “I think that we need to send out the messaging that we are safe and that this is how we are safe, and then we need to prove it. That we are a safe place to come to. And then I think even when it comes to our messages, it’s not using stock photos but finding people in the community who are going to – I wouldn’t say necessarily endorce, but can actually speak to what creates safety for them in seeking services.” (SP6)  “And again, I think that we really need to look at some foundational education, both for postsecondary educations and agencies that we need to meet. And not so much – I’ve been in the system a long time, and we used to have this mandatory, you know, 12 or 16 hours of cultural awareness training every year. That doesn’t make you culturally aware. We should be seeking to be curious and to want to learn and to you know, what are the other factors that make your agency accessible and inclusive. And how are we proving that we are that to these communities. Somehow, we have to do that. I don’t know how. But somehow, we have to do that, more than again, we have to move away from checking the boxes that yes, all staff got this in orientation, yes we did a nice little Facebook post using someone who was trans in our Facebook post. What else are we doing to actually make it a safe environment, and how do we know it’s safe? How are we testing that out?” (SP6)  “I mean we all say that we want to hire people who identify within that spectrum, who identify as Indigenous, who identify as disabled, it’s sometimes difficult to find people to fill those roles, and so I think we need to really put in a concerted effort on filling those roles with people they can identify with as well.” (SP6)  “So we go ‘ok, women, survivors of domestic violence, sexual violence, grief and loss’ or we go ‘men, IPV, sexual violence’ or whatever, but we’re not going ‘what’s this community getting?’ we need funding for this community. We need to recognize their uniqueness and then what are the services that they identify, that they need, within their own community, so – and how does that look different? Like, IPV within this community is very different than women. A woman can get more resources, or has more access to things, than a person from this community.” (SP7)  “I know that the community itself is supporting each other, but I don’t know, I think that the barrier is, is that they’re not yet welcomed into the community at large, if that makes sense.” (SP7)  “When we respond to intimate partner violence it should not be a sole mission, we can’t be the only ones who are wanting to do it, we need to partner with other organizations, we need to partner with community members” (SP8) |

**Interview Guide**

*I. Background/Demographics:*

1. Can you please tell me the year you were born? ____________

2. What city/town do you reside in? _______________

3. What is your gender? _______________

Prompts: agender, man, woman, non-binary, trans, Two Spirit, another gender or additional gender identity.

4. What is your sexuality or sexual orientation? _______________

Prompts: asexual, bisexual, gay, heterosexual (straight), lesbian, pansexual, queer, questioning/unsure, Two Spirit, another or an additional sexuality/sexual orientation.

5. Are you currently employed?

a) No ______

b) Yes, full time ______

c) Yes, part time ______

d) Yes, casual ______

6. What is the highest grade/level of education you have completed? __________________

7. What is your total annual household income (approximate)? ________________

- 1. 8. Do you identify as having a disability and/or chronic health condition?
  2. a) _____ Yes _____ No
  3. b) If yes, please specify __________________

9. With which ethno-cultural or racial group do you most identify? ________________

a) Are you a newcomer or recent immigrant (came to Canada within the last five years)? _____ Yes _____ No

10. a) What is the relationship status with your abusive partner/ex-partner?

Married ____ Separated/Divorced ____

Common-law ____ Single ____

Dating ____ Widowed ____

Other (specify) ____

*II. Experiences of Intimate Partner Violence*

In this next section, we will ask you to discuss your experiences of intimate partner violence. When recounting your experiences of violence/abuse, please do not use any names of current or former partners.

11. In whatever detail you are most comfortable with, can you please describe the nature of the violence/abuse you experienced.

- 1. 12. Experiencing violence/abuse from an intimate partner can have profound impacts. How has your partner’s/ex-partner’s violence impacted you?
  2. Probes: Emotionally/psychologically, physically/medically, spiritually, financially, employment/school, relationships with others
  3. 13. Do you believe that your identification as a diverse gender identity and/or sexual orientation has impacted your experiences of intimate partner violence?
  4. Probes: forms of IPV specific to 2SLGBTQQ+ communities (i.e., threats of being outed, misuse of pronouns, not honoring chosen name, etc.). If yes, please describe how.

*III. Experiences Seeking Help*

Many victim/survivors (but not all) seek help for the violence perpetrated against them. We’d like to ask you some questions about your experiences of seeking help.

14. Have you ever reported your experiences of intimate partner violence to an informal support (i.e., friends, family, religious leader, etc.)?

a) If yes, describe your experience.

• If yes, did you find the assistance helpful? Unhelpful? Why or why not?

• Both positive and negative experiences?

• Did you feel safe/respected during these encounters?

b) If no, what prevented you from telling anyone about the violence or seeking help?

15. Have you ever reported your experiences of intimate partner violence to a formal support agency (i.e., police, victim services, legal service, shelter, etc.)?

- 1. a) If yes, describe your experience.

• If yes, did you find the assistance helpful? Unhelpful? Why or why not?

• Both positive and negative experiences?

• Did you feel safe/respected during these encounters?

• Was the agency/organization equipped to support victims/survivors from 2SLGBTQQ+ communities?

- 1. b) If no, what prevented you from telling anyone about the violence or seeking help?
  2. 16. Seeking help can be difficult for victims/survivors who identify with a 2SLGBTQQ+ community.
  3. a) Did your gender identity and/or sexual orientation impact your help-seeking or disclosing your experiences of abuse?
  4. b) Did anything else make it difficult to disclose or seek help?
  5. c) What challenges did you face?

Probes:

• Fear of stigma, shame?

• Isolation (including geographic)

• Access to technology (i.e., poor/unreliable/unavailable internet connection, cellular service)

• Transportation barriers (i.e., lack of transportation, expensive)

• Fear of losing your privacy, confidentiality?

• Fear of racism/discrimination?

• Fear of retaliation?

• Fear of financial insecurity/lack of housing?

• Fear of losing immigration status?

• Religious or faith-based considerations

• COVID-19 pandemic

• Other challenges?

- 1. 17. Have you ever been involved with the criminal and/or family court systems because of the intimate partner violence that you’ve experienced?
  2. a) If yes, describe your experience.
  3. b) Was the court sensitive to matters specific to 2SLGBTQQ+ communities (i.e., honoring chosen name, correct use of pronouns, etc.)?

18. Did you experience intimate partner violence during the COVID-19 pandemic?

a) If yes, what issues/challenges did this present in terms of your ability to seek help?

19. Do you have any suggestions or recommendations for ways in which services for members of 2SLGBTQQ+ communities who experience intimate partner violence could be improved?

*IV. Perceptions of Intimate Partner Violence*

- 1. 20. Do you perceive intimate partner violence to be a significant problem within 2SLGBTQQ+ communities?
  2. a) If yes, why?
  3. b) If no, why not?

21. Do you feel that the issue of intimate partner violence in 2SLGBTQQ+ communities is taken seriously? Probes: by professionals, by the broader community, by other members of 2SLGBTQQ+ communities?

- 1. 22. Have you encountered any myths/misconceptions surrounding intimate partner violence in 2SLGBTQQ+ communities?
  2. a) If so, where? From whom?
  3. b) In your opinion, what are the impacts of these myths/misconceptions?

23. Is there anything else you’d like to tell me about your experiences of intimate partner violence? Is there anything else you think we should know?

*Thank you for taking the time for this interview and for sharing your thoughts and experiences. This can be a difficult topic to talk about and your responses are very valuable in helping us to understand experiences of intimate partner violence in 2SLGBTQQ+ communities.*
